# The Specificity and Flexibility of L1 Reverse Transcription Priming at Imperfect T-Tracts

**DOI:** 10.1371/journal.pgen.1003499

**Published:** 2013-05-09

**Authors:** Clément Monot, Monika Kuciak, Sébastien Viollet, Ashfaq Ali Mir, Caroline Gabus, Jean-Luc Darlix, Gaël Cristofari

**Affiliations:** 1 INSERM, U1081, Institute for Research on Cancer and Aging, Nice (IRCAN), Nice, France; 2 CNRS, UMR 7284, Institute for Research on Cancer and Aging, Nice (IRCAN), Nice, France; 3 University of Nice-Sophia-Antipolis, Faculty of Medicine, Nice, France; 4 Ecole Normale Supérieure de Lyon, Human Virology Department, INSERM U758, Lyon, France; University of Rochester, United States of America

## Abstract

L1 retrotransposons have a prominent role in reshaping mammalian genomes. To replicate, the L1 ribonucleoprotein particle (RNP) first uses its endonuclease (EN) to nick the genomic DNA. The newly generated DNA end is subsequently used as a primer to initiate reverse transcription within the L1 RNA poly(A) tail, a process known as target-primed reverse transcription (TPRT). Prior studies demonstrated that most L1 insertions occur into sequences related to the L1 EN consensus sequence (degenerate 5′-TTTT/A-3′ sites) and frequently preceded by imperfect T-tracts. However, it is currently unclear whether—and to which degree—the liberated 3′-hydroxyl extremity on the genomic DNA needs to be accessible and complementary to the poly(A) tail of the L1 RNA for efficient priming of reverse transcription. Here, we employed a direct assay for the initiation of L1 reverse transcription to define the molecular rules that guide this process. First, efficient priming is detected with as few as 4 matching nucleotides at the primer 3′ end. Second, L1 RNP can tolerate terminal mismatches if they are compensated within the 10 last bases of the primer by an increased number of matching nucleotides. All terminal mismatches are not equally detrimental to DNA extension, a C being extended at higher levels than an A or a G. Third, efficient priming in the context of duplex DNA requires a 3′ overhang. This suggests the possible existence of additional DNA processing steps, which generate a single-stranded 3′ end to allow L1 reverse transcription. Based on these data we propose that the specificity of L1 reverse transcription initiation contributes, together with the specificity of the initial EN cleavage, to the distribution of new L1 insertions within the human genome.

## Author Summary

Jumping genes are DNA sequences present in the genome of most living organisms. They contribute to genome dynamics and occasionally result in hereditary genetic diseases or cancer. L1 elements are the only autonomously active jumping genes in the human genome. They replicate through an RNA–mediated copy-and-paste mechanism by cleaving the host genome and then using this new DNA end as a primer to reverse transcribe its own RNA, generating a new L1 DNA copy. The molecular determinants that influence L1 target site choice are not fully understood. Here we present a quantitative assay to measure the influence of DNA target site sequence and structure on the reverse transcription step. By testing more than 65 potential DNA primers, we observe that not all sites are equally extended by the L1 machinery, and we define the rules guiding this process. In particular, we highlight the importance of partial sequence complementarity between the target site and the L1 RNA extremity, but also the high level of flexibility of this process, since detrimental terminal mismatches can be compensated by an increasing number of interacting nucleotides. We propose that this mechanism contributes to the distribution of new L1 insertions within the human genome.

## Introduction

Retrotransposons are highly repetitive and dispersed sequences, accounting for almost half of our DNA [1]. These elements have the ability to proliferate in genomes through an RNA-mediated copy-and-paste mechanism, called retrotransposition. LINE-1 (L1) elements are the only autonomously active elements in humans and one of the most active elements in mice. They belong to the broad family of non-LTR retrotransposons (see [2]–[6] for recent reviews).

L1 retrotransposition starts with the transcription of a 6 kb L1 RNA driven by an internal Pol-II promoter [7]. After its export to the cytoplasm, the bicistronic L1 mRNA is translated into two proteins (ORF1p and ORF2p), which associate preferentially in *cis* with their encoding mRNA [8]–[11]. This is a critical feature of the L1 replication mechanism since it limits the association of the L1 machinery with other cellular mRNAs, including defective L1 RNA sequences, and thus increases the specificity of the reverse transcription process. The resulting complex is a stable ribonucleoprotein (RNP) thought to form the core of the retrotransposition machinery [10], [12]–[19]. Its precise composition is currently unknown but it contains at least the L1 RNA and the ORF1p and ORF2p proteins [10], [16], [18], [19]. The ORF1p protein is a trimeric RNA binding protein with RNA chaperone activity [20]–[25] and the ORF2p protein shows endonuclease (EN) and reverse transcriptase (RT) activities [26], [27]. All are essential to L1 retrotransposition [16], [18], [28], [29]. The L1 RNP is imported into the nucleus where reverse transcription and integration into the host genome take place [30].

The current model for non-LTR retrotransposon integration, named target-primed reverse transcription (TPRT), was originally deduced from biochemical studies on the insect R2Bm element [31]. This retrotransposon encodes a single protein with EN and RT activities and integration of new copies occurs at a specific and defined position in the rDNA [31], [32]. The TPRT process is initiated by the formation of a nick in the genomic double-stranded DNA target. Then the R2 RT extends the newly formed 3′OH using the R2 RNA as a template [27], [31], [33]–[35]. Priming of reverse transcription occurs without any complementarity between the R2 RNA template and the DNA target site [36], [37]. Non-LTR retrotransposons can be divided into several clades, which differ considerably in the machinery that they encode (single or multiple ORFs, restriction-like or APE-endonuclease, RNaseH or not, etc…) [38]. Despite these differences, cell culture-based retrotransposition assays and analyses of novel or recent integration sites have revealed the same overall requirement for EN and RT activities, supporting the TPRT model [28], [39]–[43]. Intriguingly, non-LTR retrotransposon 3′ ends and preintegration sites often exhibit partial sequence identity, suggesting that annealing of the target site DNA to the RNA template might be a necessary step to prime reverse transcription, in contrast to R2 [40]–[43]. This step could significantly influence the genomic distribution of these elements, by imposing additional constraints after the initial endonuclease cleavage.

As regards L1, conclusive evidence on whether primer-template complementarities are required for efficient reverse transcription initiation is lacking. Most L1 pre-integration sites contain an EN recognition sequence (5′-TTTT/A-3′) and are often preceded by T-tracts of variable length [1], [27], [44]–[50]. Thus, in theory, the region covering the EN consensus and its upstream sequence has the ability to base-pair with the L1 poly(A) tail and to promote reverse transcription initiation. Nevertheless, target sites frequently contain nucleotides other than Ts, sometimes at the 3′ terminal end of the nicked DNA, which could severely impair interaction with the L1 RNA and extension by L1 RT. On the other hand, isolated recombinant L1 ORF2p produced in insect cells was found to equally extend any linear DNA substrate *in vitro*, without apparent sequence or structure requirement, or any need for primer-template complementarity [33]. Likewise, native L1 RNPs enriched from cells are able to extend oligonucleotides ending with terminal mismatches [10], [51], indicating that complementarity base-pairing between the 3′ end of the target DNA and the L1 RNA template is not an absolute requirement. But Kulpa and Moran also observed that primer sequence could influence RT initiation [10]. A common limitation of these previous studies was the use of PCR-based assays, which precluded a quantitative comparison of priming efficiencies and might lead to the detection of marginal products.

Here, we addressed the question whether - and to which degree - the liberated 3′-hydroxyl extremity on the genomic DNA needs to be accessible and complementary to the poly(A) tail of the L1 RNA for efficient priming of reverse transcription. To achieve this goal, we validated a direct L1 extension assay (DLEA) to quantitatively measure the ability of native L1 RNPs to initiate reverse transcription. Then we systematically assayed more than 65 DNA substrates varying in sequence and structure, allowing us to define the preferential rules of L1 reverse transcription priming. Our results clarify the importance of base-pairing between the L1 RNA template and the target site DNA for this process and demonstrate its exceptional flexibility.

## Results

### A direct L1 extension assay (DLEA) to study the initiation of reverse transcription by native L1 RNPs

**Figure 1:**
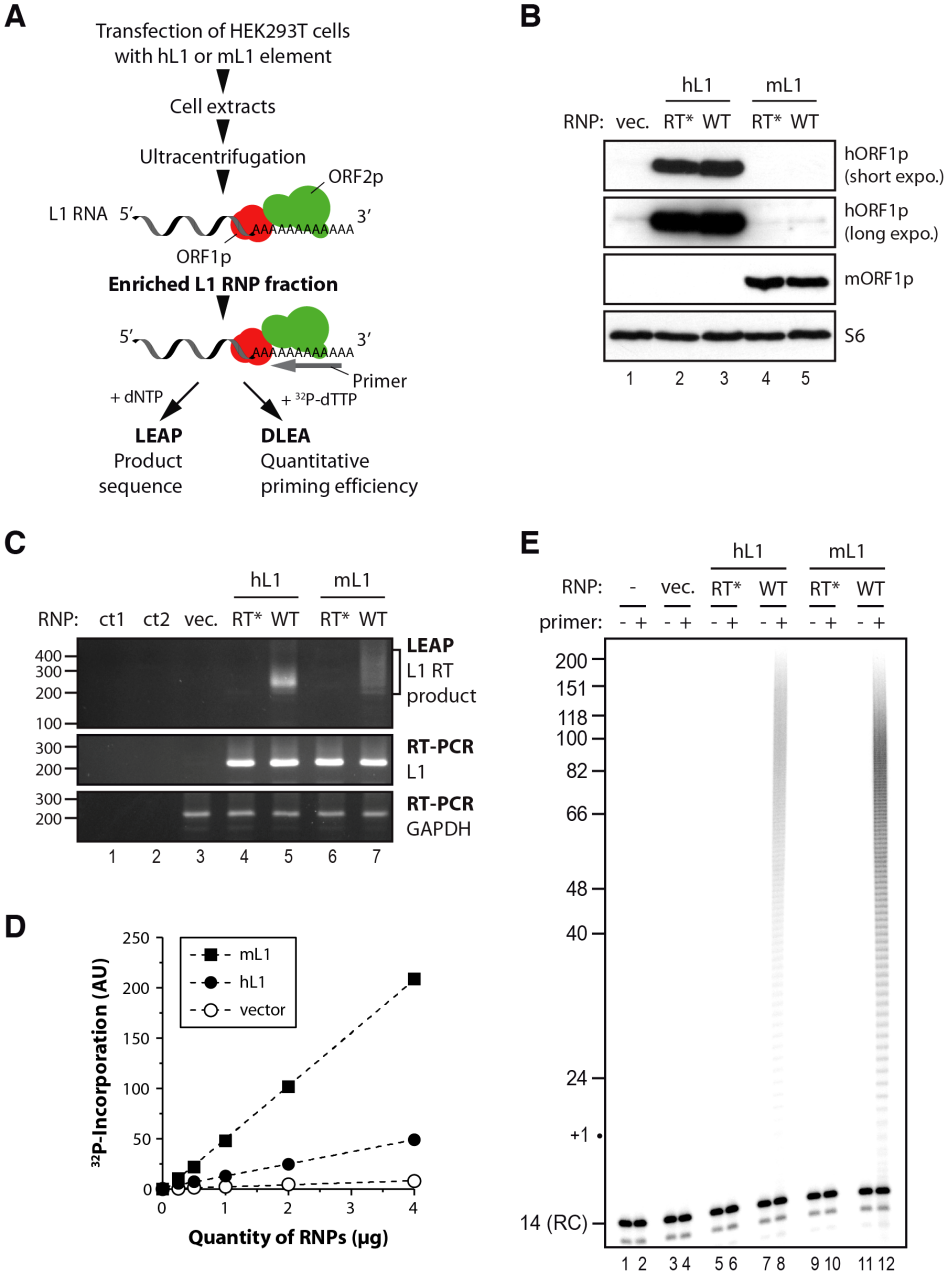
Initiation of L1 reverse transcription by native L1 RNPs. (A) Outline of the experimental procedure. LEAP, L1 element amplification protocol; DLEA, Direct L1 Extension Assay (B) Immunoblotting of human ORF1p (top 2 panels) or murine ORF1p (panel 3 from the top) in RNPs (16 µg) prepared from cells transfected with empty vector (lane 1), RT* hL1 (lane 2), wild-type hL1 (lane 3), RT* mL1 (lane 4), wild-type mL1 (lane 5). Ribosomal S6 protein was detected using an anti-S6 antibody and was used as an RNP loading control (bottom panel). (C) Detection of L1 RT activity by LEAP (top panel) and of L1 RNA by conventional RT-PCR (middle panel) in RNP preparations. GAPDH RNA is a cellular RNA used as a loading control for all RNPs (bottom panel). Annotations are the same as in (B). ct1, a control for the PCR step without cDNA; ct2, a control for the RT step without RNP or RNP-extracted RNA. The LEAP product is a diffuse smear starting from 207 bp (bracket). (D) Standard curve of murine (black square) or human (black circles) L1 RNP DNA polymerase activity, showing linear conditions, compared to vector control RNP (empty circles). Note that the intrinsic activities of mL1 and hL1 RNPs cannot be directly compared due to potential differences in their levels of expression. (E) Direct L1 extension assay (DLEA) with or without a (dT)_18_ primer in the presence of α-^32^P-dTTP (even and odd lanes, respectively). Sucrose cushion fractions prepared from human (lanes 5–8) or murine (lanes 9–12) L1-transfected cells or vector-transfected cells prepared in parallel (lanes 3–4) were used as a source of RNPs. Trace amounts of a 14-nt 5′ end-labeled oligonucleotide was added *after* the reaction as a recovery control (denoted RC). RT*, RT-defective L1 RNP; WT, wild-type L1 RNP.

To test the DNA primer requirements for initiating L1 reverse transcription, we set up a direct L1 extension assay (DLEA), which would avoid PCR and therefore would allow us to quantitate L1 priming efficiencies. The L1 retrotransposition machinery is notoriously difficult to express and to detect in most experimental systems. To obtain sufficient amounts of L1 RNPs for direct detection, we modified the protocol developed by Kulpa and Moran [10] by transiently overexpressing the canonical human L1.3 element [28] (referred thereafter as hL1) or a codon-optimized murine L1spa element (Orfeus [52], referred thereafter as mL1) in HEK293T cells, followed by a 3-day selection of transfected cells. HEK293T cells are transfected with much higher efficiency and express higher levels of transgenes than the HeLa cells, which were used in the original protocol. Then we prepared native L1 RNPs from cell extracts by sucrose cushion ultracentrifugation as previously reported (Figure 1A) [10]. In parallel, we prepared RNPs from empty vector-transfected cells or with a point mutation in the RT active site (D702A for hL1 and D709A for mL1, referred thereafter as RT* L1) as negative controls. We detected the mORF1p protein in RNP preparation from mL1-transfected cells but not from hL1 or empty vector-transfected cells by immunoblotting (Figure 1B, compare lanes 1–3 with 4–5). Similarly hORF1p levels were much higher in hL1-transfected cells than in vector control cells (Figure 1B, lanes 2–3). However long exposure revealed low levels of endogenous hORF1p in all RNP preparations (Figure 1B, lanes 1 and 4–5). To evaluate the presence of L1 RT activity and L1 RNA associated with ORF1p in the RNP preparations, we used the L1 element amplification protocol (LEAP) in which the L1 RT first extends a primer and the resulting cDNA is subsequently amplified by PCR [10]. The PCR primers are anchored in the tail of the RT primer and in the Neomycin-resistance genetic marker inserted in the transfected L1 3′ UTR. Therefore only products produced from the transfected L1 element can be amplified. Since hL1 and mL1 share the same genetic marker, the same primers can be used for both elements. As expected from previous work [10], [18], we detected L1 RT activity only in the RNP prepared from wild-type hL1 or mL1, but not in the vector or RT-defective L1 transfected cells (Figure 1C, top panel, compare lanes 5 and 7 with 3–4 and 6), even if the L1 RNA is present (Figure 1C, middle panel). Sequencing of the LEAP products confirmed that hL1 or mL1 RNA was reverse transcribed. This indicated that RNPs produced in our experimental conditions contain the core of the L1 machinery and used L1 RNA as a template. Previous studies have shown that L1 RNPs enriched on sucrose cushion as prepared here co-fractionate with many other cellular RNPs, including ribosomes [10], [16]. However, the L1 RNA is reverse transcribed at least 100 times more efficiently than other co-fractionating abundant cellular RNAs [10], a property known as L1 cis-preference [8], [9].

We reasoned that if L1 RNPs were active enough we should detect the extension of an oligo(dT)_18_ primer in the presence of radiolabelled ^32^P-dTTP. This reaction would mimic the initiation step of L1 reverse transcription, which starts at the poly(A) tail of the L1 RNA. After a 4 min incubation at 37°C, we purified the reaction products and resolved them on sequencing gels. A short end-labeled oligonucleotide was added *after* the reaction as a recovery control (RC). No or minimal extension was detected in vector or RT-defective controls consistent with the presence of only minimal amounts of endogenous hL1 activity in RNP preparations (Figure 1E, lanes 3–6 and 9–10, and Figure 1D). In contrast when wild-type hL1 or mL1 element was transfected we could easily detect the incorporation of radiolabelled dTMPs (Figure 1D and Figure 1E, lanes 8 and 12). Importantly, the amount of product formed was linearly dependent on the amount of L1 RNPs (Figure 1D), showing that the levels of primer extension could be quantitatively measured under the reaction conditions employed (linear phase, also known as initial velocity phase). We focused our work on reverse transcription initiation by using short extension times (4 min) and by adding only ^32^P-dTTP to the reaction and no other dNTP. In these experimental conditions, the products were short enough to be resolved on sequencing gels and we could follow the extension at the nucleotide resolution. The linear phase ranged from 0.2–0.25 µg up to 4 µg of RNPs, which indicates a dynamic range between 10- and 20-fold (data not shown). We chose to use 2 µg of RNPs, at the upper end of the linear range, for all following experiments and to set to 100% the level of extension obtained with an oligo(dT)_18_ primer under these conditions. Based on the dynamic range of the initial RNP titration, primer extension efficiencies as low as 5% should therefore be reliably quantified. The products are heterogeneous in length, consistent with the expected products of poly(A) reverse transcription and range from 19 nucleotides (nt) to approximately 150 nt (Figure 1E, lanes 8 and 12).

To further confirm that the ladder observed results directly from the reverse transcriptase activity of the transfected L1 element, we performed additional controls. RNase treatment reduced primer extension to undetectable levels (Figure S1A, compare lanes 2 and 3), showing that the detected DNA polymerase activity is RNA-dependent. If the reaction is conducted in the presence of RT inhibitors known to inhibit L1 retrotransposition and recombinant L1 RT activity [53]–[55] such as AZT or d4T, DNA polymerization is abolished (Figure S1B, compare lanes 2 and 3–4). No extension was detected in these experimental conditions with radiolabelled dATP, dGTP or dCTP in agreement with the reverse transcription of the poly(A) sequence (data not shown). When extension time was prolonged to 1 h (Figure S1C), the reaction was not in its linear phase anymore (and the assay was no longer quantitative). Products were longer than the maximum poly(A) length in mammals (∼250 nt), which is likely to result from L1 RT slippage in the poly(A) track as recently reported *in vivo* [56]. If all four dNTPs were present in the reaction, high molecular weight products appeared, consistent with reverse transcription ongoing beyond the L1-poly(A) boundary (Figure S1D) and in agreement with the LEAP results (Figure 1C).

Altogether these results show that DLEA detects *bona fide* initiation of reverse transcription by native mammalian L1 RNPs through the direct incorporation of radiolabeled dTMP in a primer extension reaction. Importantly, DLEA is quantitative since it demonstrates a linear relationship between the signal and RNP quantities under the reaction conditions employed.

### Efficient extension of single-stranded DNA by the L1 RNP requires at least 4 terminal matching bases

**Figure 2:**
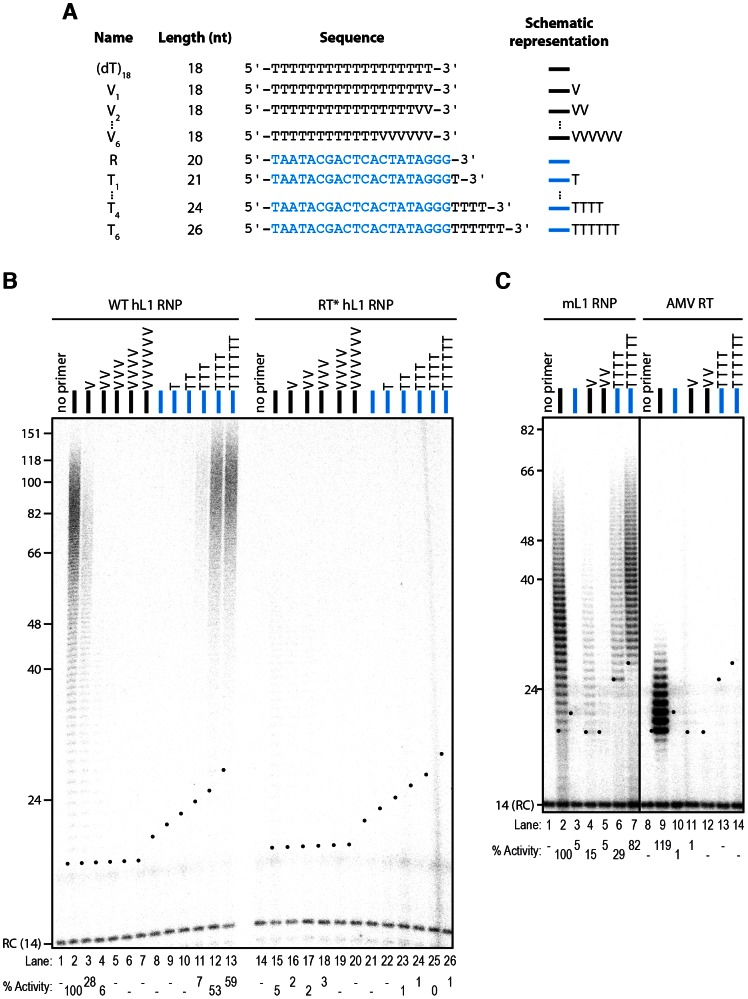
The L1 RNP preferentially extends primers ending with at least 4 Ts. (A) Scheme of the primers used. The oligonucleotide shown in blue and named R corresponds to the T7 promoter primer chosen as an unrelated sequence. V is the IUPAC nucleotide symbol for A, G or C but not T. (B) DLEA showing the extension of single-stranded primers by hL1 RNPs in the presence of α-^32^P-dTTP. (C) Comparison of the mouse L1 RNP and AMV RT for their ability to extend single-stranded primers in the presence of α-^32^P-dTTP. Experimental conditions were as in Figure 1. As a template, poly(rA) was added to the reaction performed with the AMV RT. Lanes 1–7 and 8–14 are from the same gel. RC denotes a 14 nt recovery control added after the reaction but before DNA purification. The black dots on the left side of each lane indicate the expected start of reverse transcription. Their position varies since primer length varies. Quantification of primer extension (% Activity) was relative to levels of extension obtained with oligo(dT)_18_.

In contrast to most DNA polymerases, it was previously demonstrated that the hL1 RNP is able to extend a terminal mismatched base pair using a PCR-based assay followed by sequencing of the products [10]. To determine more quantitatively the efficiency of extension of such mismatched primers, we changed the last nucleotides of the oligo(dT)_18_ primer to a non-T nucleotide in order to prevent base-pairing of the primer 3′ end to the L1 poly(A) tail (Figure 2A). Although decreased as compared to the oligo(dT)_18_ primer, the hL1 RNP can extend a primer with a single or double terminal mismatch (V_1_ and V_2_, Figure 2B, lanes 3–4; V = not T) or with a mismatch at the penultimate position (VN, 15% of the oligo(dT)_18_ extension, not shown), in agreement with previous reports [10], [51]. In contrast, if the primer ends with more than two mismatched nucleotides (V_3_ to V_6_), DNA polymerization becomes undetectable under the employed reaction conditions (Figure 2B, lanes 5–7). Similarly, the hL1 RNP is not able to efficiently use an unrelated oligonucleotide ending with three Gs (the T7 promoter primer, noted R, Figure 2A) as a primer for its reverse transcription (Figure 2B, lane 8).

**Figure 3:**
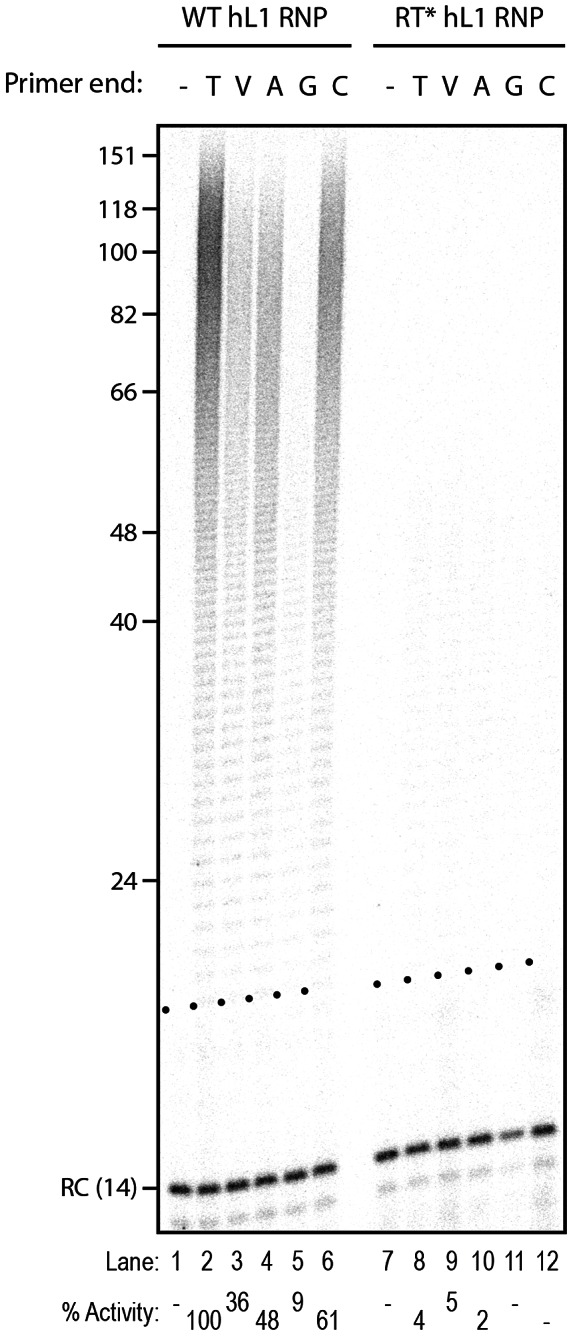
Influence of the terminal nucleotide on primer extension by L1 RNP. DLEA showing the extension of single-stranded primers by hL1 RNPs in the presence of α-^32^P-dTTP. All primers are oligo(dT)_17_-X oligonucleotides, where X corresponds to the nucleotide indicated above the lanes. V is the IUPAC nucleotide symbol for A, G or C but not T. (−) is a control without primer. Experimental conditions were as in Figure 1. RC denotes a 14 nt recovery control added after the reaction but before DNA purification. The black dots on the left side of each lane indicate the expected start of reverse transcription. Quantification of primer extension (% Activity) was relative to levels of extension obtained with oligo(dT)_18_ (lane 2).

Next, we measured the influence of each individual terminal base on primer extension. Although all terminal mismatches reduced the efficiency of reverse transcription initiation to some extent, a terminal G was the most detrimental, whereas a C or an A was better tolerated (Figure 3). Thus the levels of extension of a T-tract is dependent on the nature of its 3′ terminal base with the following preference: T>C>A>G.

To further characterize the need for terminal matching nucleotides in the priming of hL1 reverse transcription, we added an increasing number of Ts to the R primer (T_1_ to T_6_). Initiation of reverse transcription is robustly detected only when the single-stranded primer ends with at least 4 Ts and trace activity can already be detected with 3 terminal Ts (Figure 2B, lanes 11–13). We obtained similar results with mL1 RNPs (Figure 2C, lanes 1–7 and Figure S2).

In order to compare the properties of the native L1 RNPs with a retroviral RT, we tested the ability of recombinant Avian Myeloblastosis Virus (AMV) RT to prime reverse transcription under identical experimental conditions. In these experiments, exogenous poly(rA) was added as a template together with quantities of the AMV RT that lead to similar levels of extension as the L1 RNP using the (dT)_18_ primer (Figure 2C, compare lanes 2 and 9). Under these experimental conditions, reverse transcription by AMV RT was not primed by oligonucleotides ending with terminal mismatches (Figure 2C, compare lanes 4–5 to 11–12) or by oligonucleotides ending with 4 or 6 Ts (Figure 2C, compare lanes 6–7 to 13–14). These observations suggest that limited base-pairing interactions between the primer and the template might be stabilized by the L1 RNP, through direct binding of ORF1p or ORF2p to the single-stranded DNA. In addition, the extension products of the (dT)_18_ oligonucleotide obtained with the AMV RT are much shorter than those obtained with the L1 RNP. This might suggest that the L1 RNP is more processive than the AMV RT and/or that the L1 RNP has a higher affinity for dTTP than AMV RT as shown for the R2 element [57], [58]. However, since the templates used are not strictly similar, it is difficult to draw definitive conclusions on this aspect.

It was previously reported that a nuclease activity in the RNP preparations could process primers before their extension [51]. Thus, in principle, it is possible that primers ending with terminal mismatches are first processed to eliminate the mismatch(es) and then extended. Against this possibility, the majority of the products observed in sequencing gels start at the expected +1 position or above (Figure 2 and Figure S2). As an additional control, we performed LEAP reactions using primers ending with the same sequence as depicted in Figure 2A. We could amplify, clone and sequence products with up to 3 terminal mismatches (Figure S3A). Although a small percentage of processed primers were found (7 out of 160 sequences in total), the majority of the mismatches were directly extended (Figure S3C). Thus differences of extension are not due to differential processing of the primers. We note that the levels of the nuclease activity responsible for primer processing, which co-fractionates with L1 RNPs in sucrose gradients, might dependent on the cell type used to prepare RNPs. Using the same RACE primer ending with VN, Kulpa *et al*. observed processing in 33/81 (39%) of the analyzed clones obtained with HeLa cells, while Kopera *et al.* found 5/45 (11%) of processed primers in CHO-derived cell lines. In comparison, we obtained 2/70 (3%) clones showing a processed primer with RNPs prepared from HEK293T cells.

Altogether these observations show that native L1 RNPs efficiently prime reverse transcription at DNA ending with 4–6 terminal matching nucleotides, although it can accommodate terminal mismatches with lower priming efficiencies.

### The L1 RNP extends primers mimicking *bona fide* insertion sites with variable efficiencies

**Figure 4:**
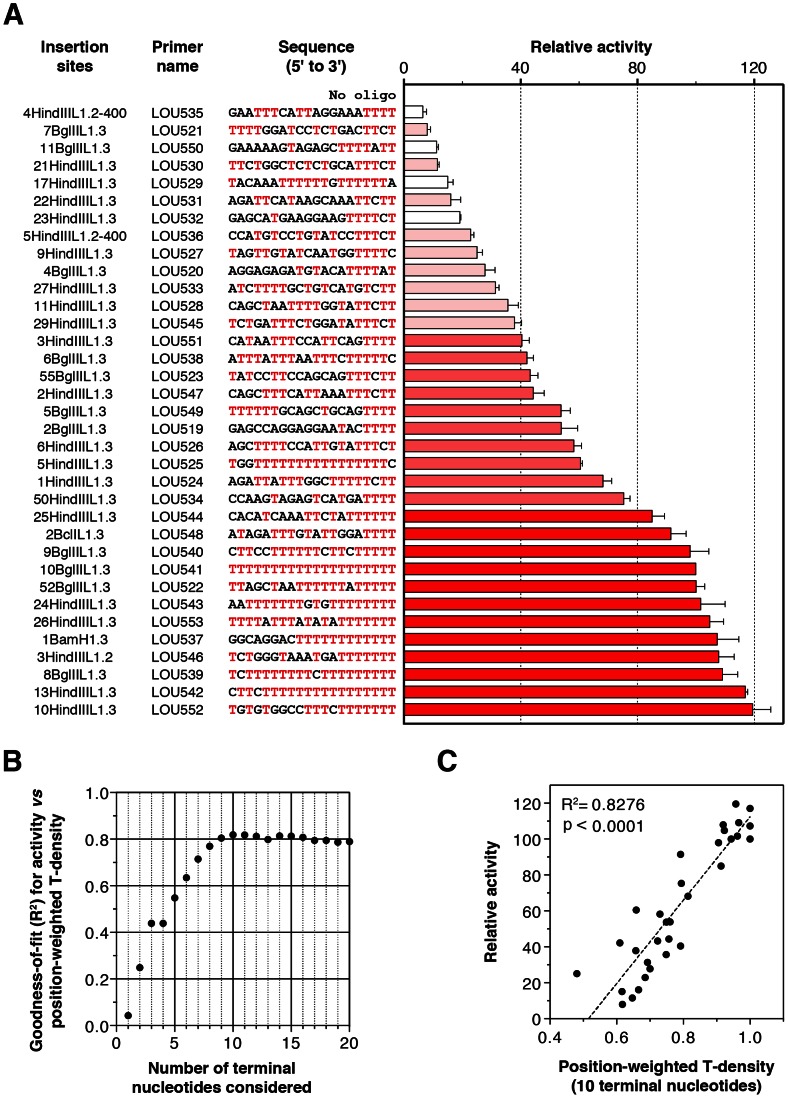
Extension of primers mimicking *bona fide* human L1 insertion sites by the human L1 RNP. (A) Relative extension of primers as measured by DLEA. Extension of each primer was normalized to the extension levels obtained with the (dT)_20_ primer (LOU541 corresponding to the 10BglIIL1.3 insertion site). This ratio, expressed as a percentage, was designated as ‘Relative activity’. Bars were color-coded and sorted according to the efficiency of priming (red, activity ≥80%; medium red, 40%≤Activity<80%; light red, activity <40%; white, primers excluded from the correlation analyses due to hairpin formation). Bars indicate the mean and error bars the S.E.M. (n = 3). The name of the insertion sites correspond to the recovered clones from cultured cells published in [46]. (B) A role for the primer terminal nucleotides in hL1 RNP reverse transcription priming. For each *n* between 1 and 20, the correlation between activity and position-weighted T-density of the terminal *n* nucleotides was calculated. The goodness-of-fit (R^2^) only marginally changes when *n*>10, indicating that the terminal 10 nucleotides are the most relevant determinants for priming efficiency. Note that the 4^th^ bases at the 3′ terminus in all the primers of this set are coincidentally identical (T). For this reason, R^2^ is identical for *n* = 3 and *n* = 4. See the ‘Results’ and ‘Material and Methods’ sections for a detailed definition of the position-weighted T-density. (C) An example of correlation between the density of Ts close to the 3′ end of the primer (position-weighted T-density) and the efficiency of reverse transcription priming (for *n* = 10). For the graph shown in (B) and (C), primers which could fold into a structured hairpin (white bars in A) were excluded from the analysis (see Figure 6, Figure 7, Figure 8 for a detailed analysis of primer structure on reverse transcription efficiency).

L1 EN-mediated nicking at a consensus target site produces a 3′-OH DNA ending with four Ts [27], [44]. This is consistent with our observation that the L1 RT can extend primers ending with as little as four Ts. However, L1 integration sites often contain degenerate L1 EN recognition sites that differ from the consensus recognition sequence [1], [46], [47]. This prompted us to analyze the ability of native hL1 RNPs to extend primers which mimic *bona fide* insertion sites. We designed 35 primers corresponding to previously published insertion sites recovered from new hL1 retrotransposition events obtained in cultured cells [46]. The sequence and the original name of each recovered clone is indicated in Figure 4A. Levels of extension were normalized to those obtained with the primer LOU541 (clone 10BglIIL1.3), which corresponds to a (dT)_20_ oligonucleotide.

We observed that all sites are not equally extended (see Figure 4A). The levels of extension range between 7% (LOU535) and 120% (LOU552). The best primer is 17-fold more extended than the least-efficient primer. Even if we know that these target sites were used *in vivo* without processing [46], we choose six of them differing from each other by the position or the nature of the mismatched nucleotides to perform LEAP (Figure S3B) and we sequenced the products. Again we found a small number of processed primers (∼5%), but the majority of products result from the direct extension of mismatched primers (Figure S3).

We categorized primers based on their potential of extension (Figure 4A; 0–40%, light red; 40–80%, medium red; 80–120%, dark red). Four primers have the ability to form stable hairpins (Figure 4A, white bars), and were excluded from further analyses since hairpin formation is dependent on primer length, which was arbitrarily chosen (the specific impact of primer structure on L1 RT initiation is presented at the end of the ‘Results’ section). Top ranking primers (dark reds) all end with at least 4 Ts, often more, and are extremely rich in Ts, in agreement with the results presented in Figure 2. Interestingly, primers with a mismatch in the last critical four nucleotides are more efficiently extended if they are preceded by a T-rich upstream sequence. For example, primers LOU525, LOU527 and LOU538 all end with 5′-TTTC-3′ and their respective levels of extension are LOU527<LOU538<LOU525, which roughly follows the number of Ts close to the 3′ end. This suggests a compensation mechanism allowing the extension of primers ending with suboptimal sequences.

To address the significance of this phenomenon more quantitatively, we calculated for each oligonucleotide two parameters: (i) the density of Ts (number of Ts/length of the oligonucleotide), which simply reflects the abundance of Ts in the primer, and (ii) the position-weighted T-density, which is similar but the weight of each T is inversely proportional to the distance from the 3′ end (see Material and Methods section for more details). Using linear regression, we found that the activity correlates significantly with both parameters (p = 0.0002 and p<0.0001, respectively) but the goodness-of-fit is much better with the position-weighted T-density than with the T-density (R^2^ = 0.7895 *vs* 0.3950, not shown). To evaluate the number of terminal nucleotides that contribute to priming efficiency, we further correlated the priming efficiency with position-weighted T-density, taking into account a variable number of terminal nucleotides. The goodness-of-fit (R^2^) increases steadily up to 10 considered nucleotides and then reaches a plateau (Figure 4B). Considering nucleotides beyond position 10 (from the 3′ primer end) does not improve the correlation. The correlation between priming efficiency and the position-weighted T-density when only the last 10 nucleotides are considered is plotted in Figure 4C (R^2^ = 0.8276).

In conclusion, we have demonstrated biochemically that complementarity between the L1 poly(A) tail and the last 10 nucleotides of the target DNA plays a role in extension at the target site, the last 4 nucleotides being the most critical. Suboptimal primers with a mismatch in their last 4 nucleotides are extended with a lower efficiency, which can be partially compensated by increasing the number of Ts in the upstream sequence.

### The “snap-velcro” model and supportive evidence

**Figure 5:**
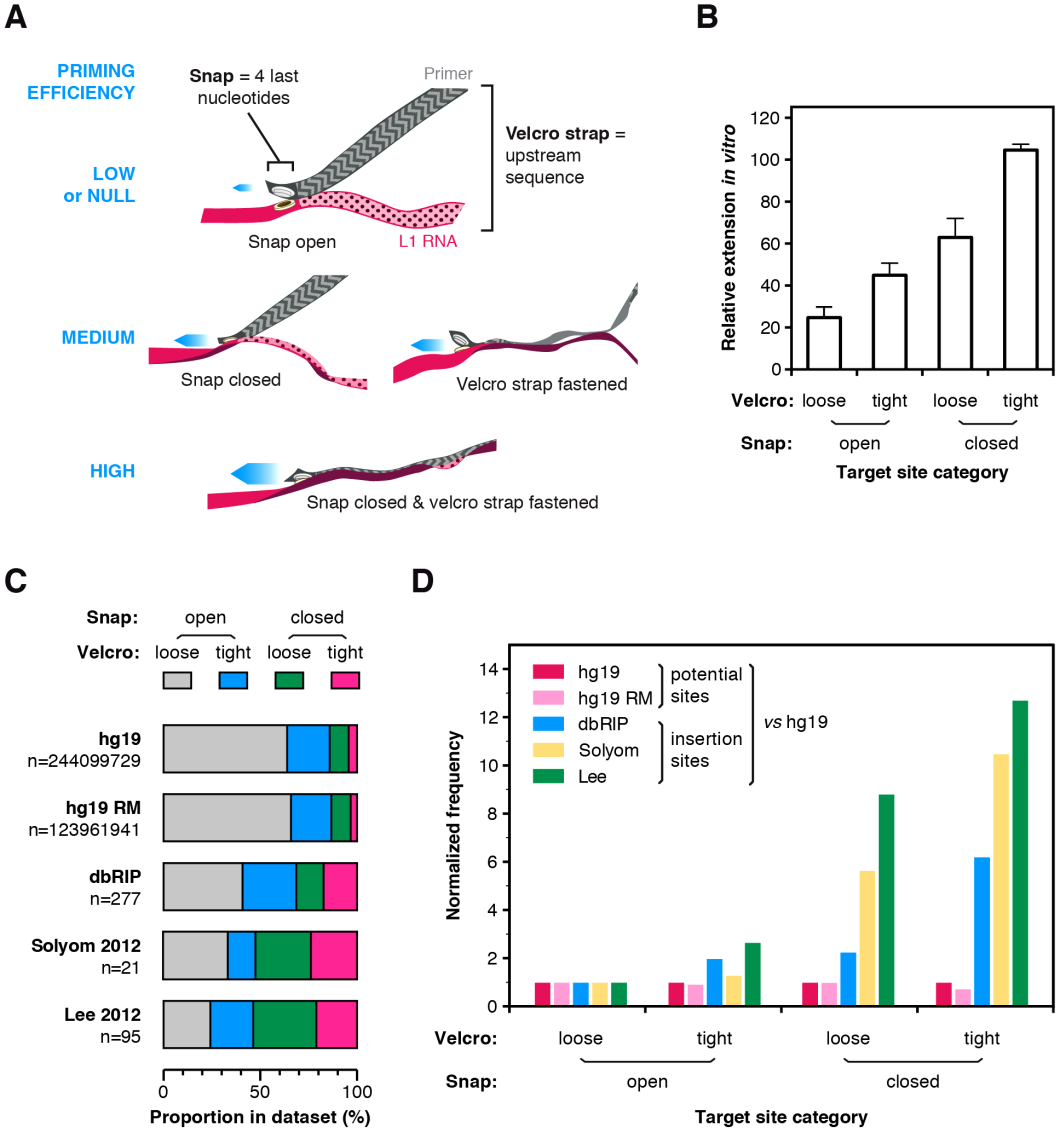
The snap-velcro model and supporting biochemical and genomic evidence. (A) A snap-velcro model for priming of L1 reverse transcription. The snap represents the 4 last nucleotides of the primer. It is considered as closed if it ends with 4 Ts (perfect terminal match) and as open if it contains a mismatch in the last 4 Ts. The velcro represents the 6 upstream bases. It is considered as tightly fastened only if the position-weighted T-score of this region is at least 50% of the maximum score. Otherwise, it is considered as loosely or not fastened. When the snap is closed and the velcro is tightly fastened, reverse transcription is high (bottom). If the snap is open *or* if the velcro is loosely fastened, reverse transcription priming is reduced (middle). Finally, if the snap is open *and* the velcro loosely fastened, reverse transcription priming is low or null (top). (B) *In vitro* efficiency of reverse transcription priming by the hL1 RNP depending of the snap and velcro status. Bars indicate the mean and error bars the S.E.M. Data are from Figure 4A, white bars excluded (see legend Figure 4). Both snap and velcro contribute extremely significantly to the differences of extension between primers (p<0.0001, two-way ANOVA). (C) Proportion of sites in the snap and velcro categories for the human genome (hg19), the repeat-masked human genome (hg19 RM) and in polymorphic L1 insertion datasets (dbRIP, Solyom 2012 and Lee 2012). Note that the proportion of sites falling in each of the snap-velcro category is significantly different in the L1 insertion datasets (dbRIP, Solyom 2012 and Lee 2012) as compared to the proportions found in hg19 or repeatmasked hg19 (Chi-square test, two-tailed P<0.0001). (D) Human L1s preferentially insert into target sites with snap closed and velcro fastened. Potential (hg19 or hg19 RM) or real (dbRIP or Lee 2012) target sites with a recognizable EN target sequence were categorized based on their snap and velcro states. The frequency of each category for each dataset was calculated and divided by the frequency of the corresponding category in the reference genome hg19 (enrichment). For each dataset, enrichment was further normalized to the enrichment of the “open snap/loose velcro” category to evaluate the respective effect of the snap and/or velcro on L1 insertion site frequencies (normalized frequency). The raw data for panels C and D are compiled in Table S2.

To illustrate these findings, we propose that the four terminal bases of the primer, which overlap with the EN nuclease recognition sequence, act as a specific snap and the upstream six bases act as a weaker velcro strap (Figure 5A). When the snap is closed (perfect terminal matches, EN consensus sequence), initiation is efficient, but is enhanced if the velcro strap (upstream bases) is also tightly fastened. Inversely, if the snap is open (terminal mismatches), extension occurs preferentially if this is compensated by a tightly fastened velcro strap. The rational to distinguish snap and velcro regions is to highlight the preponderant role of the terminal nucleotides, which is also reflected in the position-weighted T-density mode of calculation.

To test this model, we determined for each primer whether the snap is open or closed and whether the velcro strap is loosely or tightly fastened. A snap was considered closed only if the 3′ end of the primer was (T)_4_. The velcro strap was considered as tightly fastened if the position-weighted T-density score of this region was at least half of its maximum value (see Materials and Methods section for the precise definition of these states). Then for each group we calculated the mean efficiency of extension by the hL1 RNP (Figure 5B, data from Figure 4A). In agreement with the model, tightly fastened velcro improves the extension of target sites with a snap closed and partially rescue those with a snap open. Both snap and velcro contribute extremely significantly to the differences of extension between primers (p<0.0001, two-way ANOVA).

A testable prediction of this model is that, *in vivo*, at the genomic level, L1 elements would more frequently insert at putative EN recognition sites with a closed snap and a tightly fastened velcro strap; and that a tightly fastened velcro would favor insertions as compared to similar sites with an open velcro. To test this model, we searched in the human reference genome (hg19) for the position of all potential EN targets: R/TTTT, which corresponds to a closed snap; or R/VTTT, R/TVTT, R/TTVT and R/TTTV, which correspond to open snaps (R = purine, V = not T). For each of them, we extracted the 10 nucleotides upstream of the nick position and categorized each on the basis of its snap/velcro status to obtain the exact frequency of each category in hg19. Then we extracted the exact insertion sites for all the L1HS polymorphic insertions present in dbRIP [59] or in recent catalogs of somatic L1 insertions in cancer genomes [60], [61] for which the insertion sites are annotated at nucleotide resolution. Since some insertions occurred through an EN-independent mechanism, we only kept sites with a recognizable EN target (R/TTTT, R/VTTT, R/TVTT, R/TTVT, R/TTTV, as above). We categorized these sites based on their snap/velcro status. First, we determined the distribution of these categories in the human reference genome (hg19, Figure 5C) or its repeat-masked counterpart (hg19 RM, Figure 5C) and we compared it to that of L1 insertions in each dataset (dbRIP, Solyom and Lee, Figure 5C). Strikingly, the proportion of L1 insertions in sites with closed snap and/or tightly fastened velcro was significantly increased as compared to their proportion in the human genome (Chi-square test, p<0.0001 for all insertion datasets). As an additional analysis, we calculated the frequency of each category in a given L1 insertion datasets as compared to their frequency in the human genome. We normalized this enrichment relative to the insertion sites with an open snap and a loosely fastened velcro strap. As shown in Figure 5D, L1 insertions are more frequent at sites with a closed snap or a tightly fastened velcro, and even more frequent at sites having both. Consistent with the *in vitro* data, given a snap status, insertions are more frequent at sites with a tightly fastened velcro than with a loosely fastened velcro. Other studies have previously reported that T-richness extends beyond four nucleotides upstream of the cleavage site [48], [50]. Our analysis differs from these previous observations in that each position is not considered independently from the others. Altogether the distribution of polymorphic L1 insertions *in vivo* is consistent with the snap-velcro model at the genomic level, but it should also be stressed that, *in vivo*, other determinants are likely to influence L1 insertion profiles.

### Extension of dsDNA by the L1 RNP

**Figure 6:**
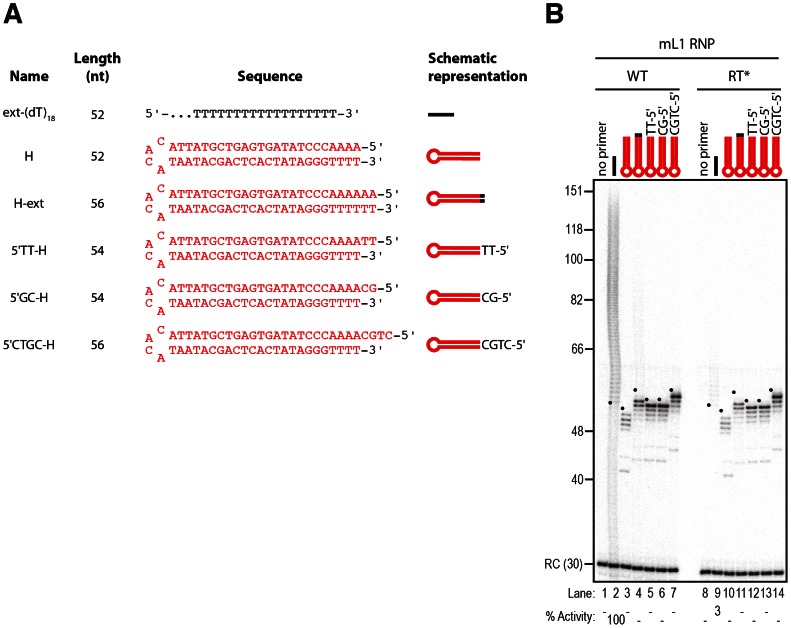
Double-stranded primers with blunt or 3′-recessed are not efficiently extended by mL1 RNPs. (A) Scheme of the primers used. (B) DLEA showing the absence of extension of double-stranded primers with blunt or 3′ recessed ends in the presence of α-^32^P-dTTP. Note that the only products observed with hairpin primers (lanes 3–7) result from contaminating cellular activities (see text and Figure 8 for further characterization). RC denotes a 30 nt recovery control added after the reaction but before DNA purification. Quantification of primer extension (% Activity) was relative to levels of extension obtained with ext-(dT)_18_ (lane 2). The black dots on the left side of each lane indicate the expected start of reverse transcription. Their position varies since primer length varies. Results obtained with hL1 RNPs were identical and are shown in Figure S4.

An alternative pathway of L1 integration uses preformed double-stranded DNA lesions instead of EN-mediated cleavage. To determine whether the L1 RNP is able to directly initiate reverse transcription at blunt DNA ends, we designed model hairpins ending with four or six Ts at their 3′ terminus (Figure 6A, primers H and H-ext). Notably, we used hairpins instead of two separate DNA strands to exclude the possibility that remaining free single-stranded primers could be extended (Figure 6A).

**Figure 7:**
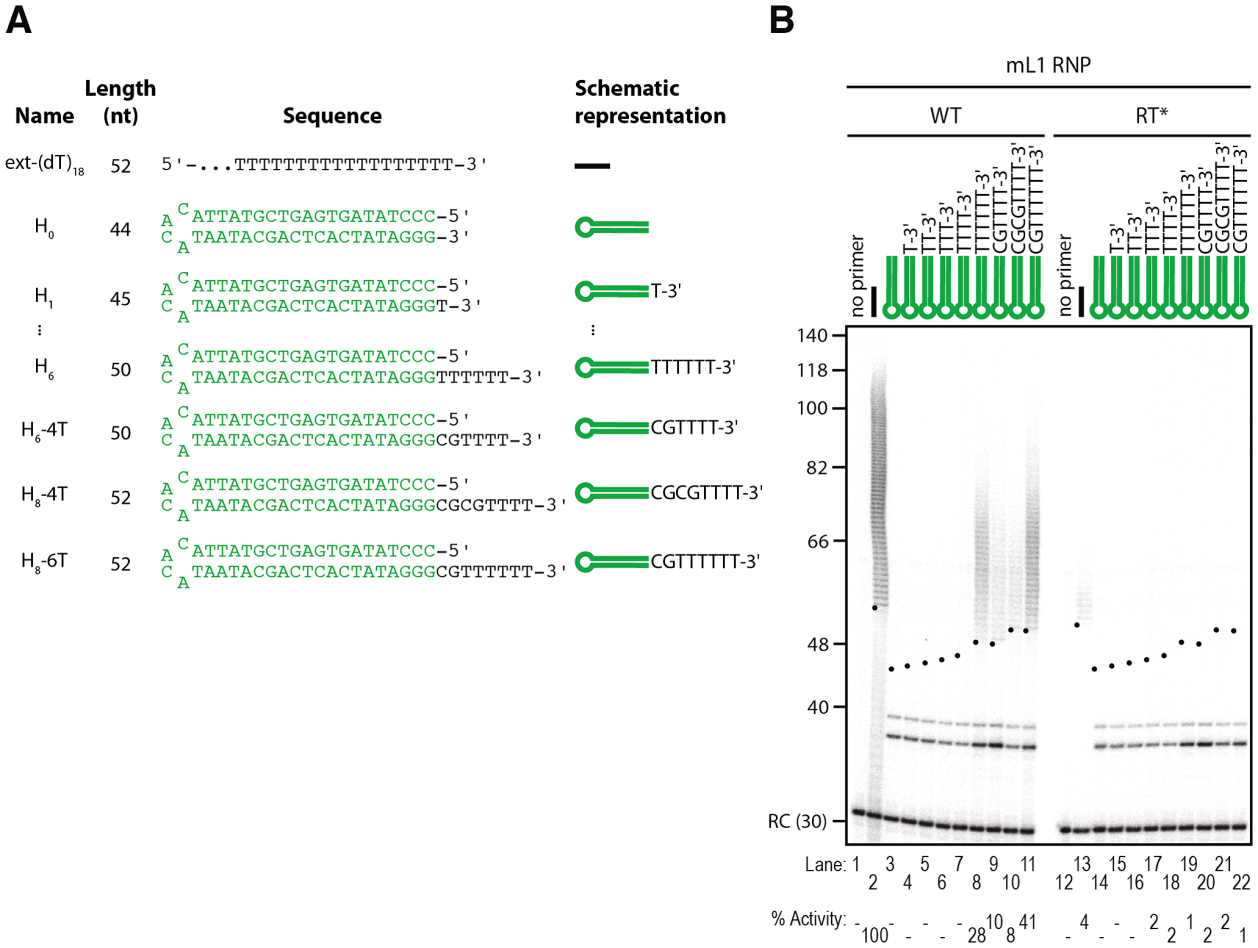
The L1 RNP preferentially extends double-stranded DNA with a 3′ overhang. (A) Scheme of the primers used. (B) Extension by mL1 RNPs of double-stranded primers ending with a 3′ overhang in the presence of α-^32^P-dTTP. Note that the doublet below 40 nt observed in lanes 3–11 and 14–22 results from contaminating cellular activities (see text and Figure 8 for further characterization). RC denotes a 30 nt recovery control added after the reaction but before DNA purification. Quantification of primer extension (% Activity) was relative to levels of extension obtained with ext-(dT)_18_ (lane 2). The black dots on the left side of each lane indicate the expected start of reverse transcription. Their position varies since primer length varies. Results obtained with hL1 RNPs were identical and are shown in Figure S4.

The expected start position of each extension product (+1), which depends on primer length (see Figure 6A), is indicated by a black dot on the left side of each lane. Although we can readily detect elongation of the single-stranded ext-(dT)_18_ primer (Figure 6B, lane 2), no mL1-specific extension was observed with these blunt substrates (Figure 6B, compare lane 2 to 3–4). The radiolabeled molecules detected below the +1 of the reverse transcription (Figure 6B, between 40 and 56 nt and Figure 7B, below 40 nt) result from contaminating activities, which co-fractionate with the mL1 RNP in the sucrose cushion (see below for a detailed characterization). In addition, we asked whether the mL1 RNP could access and extend a stretch of 4 Ts embedded in a duplex DNA. No extension was observed when we used various hairpins with 3′ recessed ends ending with 4 Ts (Figure 6A, 5′TT-H, 5′GC-H, 5′CTGC-H and Figure 6B, compare lanes 5–7 to 12–14). Identical results were obtained with hL1 RNPs (Figure S4A).

Since L1 elements are believed to integrate into double-stranded genomic DNA and L1 RNPs can efficiently extend single-stranded oligonucleotides (see above), we reasoned that L1 RNPs might be able to prime DNA synthesis on double-stranded primers ending with a 3′ overhang. To test this hypothesis we designed model hairpins extended by a 3′ overhang of increasing size (Figure 7A, primers H_0_ to H_6_). In contrast to reactions performed with blunt or 3′-recessed hairpin substrates, initiation of mL1 reverse transcription is easily detected as soon as the 3′ overhang reaches a length of 6 nt, as shown by the mL1-specific ladder which appears above 50 bp (Figure 7B, compare lane 8 to 3–7 and 19). Increasing the length of the overhang to 8 nt slightly increases the levels of reverse transcription, which indicates that a 6 nt 3′ overhang is necessary and sufficient for efficient extension by the mL1 RNP. In the experiments using single-stranded substrates, we demonstrated that 4 matching bases at the 3′ end of the substrate are sufficient to prime reverse transcription at detectable levels. This is also true for 3′ overhang hairpins, since a hairpin with a 6- or 8-nucleotide 3′ overhang but ending with only 4 Ts is extended, although to lower levels than a similar single-stranded primer ending with 4Ts (Figure 7B, lanes 9–10 and Figure S2, lane 12). Identical results were obtained with hL1 RNPs (Figure S4B).

**Figure 8:**
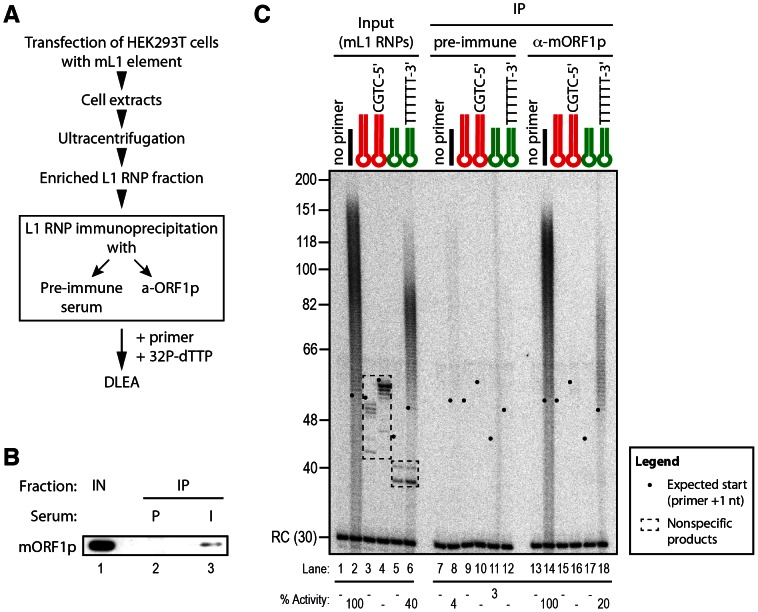
Priming of reverse transcription by immunopurified mL1 RNP. (A) Outline of the experimental procedure. (B) Immunoblot of the mL1 RNP immunoprecipitation (IP). IPs were performed on mL1 RNP preparations (Input, IN, lane 1) using preimmune (P, lane 2) or mORF1p-immune (I, lane 3) sera. Blot was probed with the anti-mORF1p serum. (C) Primer extension assay performed with mL1 RNPs (lanes 1–6), beads of the preimmune serum IP (lanes 7–12) or beads from the anti-mORF1p serum IP (lane 13–18). Note that the products suspected to be nonspecific (dashed boxes, lanes 3–6) indeed result from contaminating cellular activities and disappear upon immunoprecipitation, while the specific reverse transcription products are still observed (lanes 14 and 18). RC denotes a 30 nt recovery control added after the reaction but before DNA purification. Quantification of primer extension (% Activity) was relative to levels of extension obtained with ext-(dT)_18_ (lane 2 for Input and lane 14 for IP). The black dots on the left side of each lane indicate the expected start of reverse transcription. Their position varies since primer length varies.

As mentioned above, incubation of L1 RNP fractions with hairpin primers and ^32^P-dTTP results in labeled products, which are shorter than the expected +1 of the reverse transcription reaction (Figure 6B and Figure S4A, between 40 and 56 nt and Figure 7B and Figure S4B, below 40 nt). These products are also detected at similar levels with RT-defective L1 RNP preparations (Figure 6B, lanes 9–14 and Figure 7B, lanes 14–22) and with RNPs prepared from vector-transfected cells (data not shown), suggesting that they result from contaminating cellular activities, which co-fractionate with the L1 RNP in the sucrose cushion. To verify this hypothesis, we further purified the mL1 RNPs by immunoprecipitation using an antibody raised against the mORF1p protein (Figure 8A and 8B), and then we performed reverse transcription reactions on the beads. As a negative control, we performed the immunoprecipitation with the preimmune serum. First, we could directly detect the mL1 RT activity in the immunoprecipitated complex (Figure 8C, compare lanes 8 and 14), reinforcing the notion that the L1 RNA, ORF1p and ORF2p form a stable complex [18]. Second, the immunopurified mL1 RNP extends the H_6_ hairpin primer with a 3′ overhang but not the blunt or 3′-recessed primers (Figure 8C, compare lanes 9–12 and 15–18). Third, the short products formed upon incubation with the sucrose cushion mL1 RNP preparation disappear if the mL1 RNP is further purified by immunoprecipitation (Figure 8C, compare lanes 3–6, dashed boxes, and 15–18). Altogether these observations confirm that the bands below the +1 are indeed nonspecific products resulting from cellular contaminating activities and that the ladder-like products above ∼50 nt are *bona fide* L1 RNP reverse transcription products.

Based on these data we conclude that native L1 RNPs preferentially extend DNA substrates ending with at least 4 Ts and a 6-nt single-stranded 3′ overhang, but does not efficiently extend blunt or 3′-recessed double-stranded DNA substrates.

## Discussion

Although L1 elements are responsible for a very large part of mammalian genomes and are an important source of genetic diversity and diseases [60], [62]–[66], detailed molecular mechanisms of their replication remain poorly studied at the biochemical level. We have developed here a direct L1 extension assay (DLEA) to explore the impact of primer sequence and structure on reverse transcription initiation by native L1 RNPs (Figure 1 and Figure S1). The DLEA protocol differs from previous approaches [10], [33], [51], [55], [67] because it combines native L1 RNP purification from cell extracts, by sucrose cushion ultracentrifugation or immunopurification (Figure 8), with the direct detection of extension products. Since it does not require a PCR amplification step, the DLEA allows quantitative comparisons of priming efficiencies for a large variety of substrates with different sequences and structures. A limitation of this assay is the absence of sequence information on the product. Therefore we complemented DLEA data with LEAP amplification and sequencing.

By testing more than 65 different primers, including many that mimic *bona fide* L1 insertion sites recovered from cultured cells, we could define the rules of L1 reverse transcription initiation with an unprecedented resolution: (i) partial sequence complementarity between the 10 terminal nucleotides of the target site and the L1 RNA poly(A) tail impact reverse transcription initiation (Figure 2 and Figure S2, and Figure 4); (ii) four terminal Ts are sufficient to promote efficient extension of the target DNA (Figure 2 and Figure S2); (iii) the L1 RNP can tolerate a mismatch in the crucial last 4 nucleotides if it is compensated by an increased number of matching nucleotides upstream of these bases (Figure 2, Figure S2 and Figure 4); (iv) the preferred terminal base is T>C>A>G (Figure 3). Based on these quantitative data, we propose a ‘snap-velcro’ model to illustrate the high level of flexibility of the L1 RNP toward primer use (Figure 5A). This model identifies two distinct regions in the cleaved target DNA: (i) the terminal 3′ four nucleotides (snap), which correspond to the EN recognition site, and are also essential to reverse transcription initiation; and (ii) the upstream six nucleotides (velcro), which enhance reverse transcription efficiency and compensate potential mismatches in the snap region, when rich in Ts.

Studying the properties of L1 RNPs *in vitro* provides detailed molecular insights into specific steps of the retrotransposition process. This is a useful complement to retrotransposition cellular assays, which offer a more global view of this mechanism. Nevertheless, a number of differences between the *in vitro* and *in vivo* situations, and between endogenously and ectopically expressed L1, should be emphasized. First, reverse transcription initiation is uncoupled from the cleavage of the target DNA, in primer extension assays such as LEAP or DLEA. Thus, we cannot completely exclude that L1 RNPs would utilize a different priming mechanism in the context of a L1 TPRT reaction. Likewise, it is possible that the detected activity results from a minor fraction of the RNPs, which can only extend exogenous primers. This situation is reminiscent of L1 reverse transcription initiation at existing DNA lesions as hypothesized for EN-independent integration events [51], [68]–[70]. Second, due to read-through transcription, L1 RNAs expressed from endogenous loci sometimes contain a first poly(rA) sequence, which is transcribed by RNA-Polymerase II from the L1 poly(dA) tail and can occasionally be imperfect, followed by a downstream genomic sequence, and ending with a perfect poly(rA) tail generated by Poly(A)-Polymerase [71], [72]. Theoretically, alternative nucleotides present in such internal and imperfect poly(A) sequences could match perfectly to degenerate endonuclease sites, such that mismatches between primer and template would be less frequent. In contrast, L1 RNA polyadenylation in ectopically expressed constructs is generally driven by the strong SV40 polyadenylation sequence and by Poly(A)-Polymerase leading to perfect poly(rA) tails. Finally, our data suggest that target site choice is dictated not only by the specificity of the first EN cleavage, but also by the efficiency of RT priming after nicking. Interestingly, an engineered L1 endonuclease with relaxed sequence specificity *in vitro* has been described [73]. *In vivo*, L1 elements carrying this endonuclease variant still integrate in extended T-rich sequences, which shows that additional factors other than the EN specificity contribute to L1 insertion profile *in vivo*. Our data suggest that primer-template complementarity might be one of these factors, by promoting the initiation of reverse transcription, but it is also very likely that additional partners or inhibitors influence L1 targeting *in vivo*, modulating or relaxing EN or RT specificity. Indeed, L1 insertions occasionally take place at sites that do not strictly follow the rules described here (Figure 5C, and [46], [47], [49], [51], [69]), suggesting that primers for which we cannot detect extension by DLEA might actually be L1 substrates. From our data we can only conclude that they are extended *in vitro* at least 10–20 fold less efficiently than the best target sites that were used as references in our assays.

In contrast to the L1 RNP, R2 reverse transcriptase does not require sequence matching to prime DNA synthesis and does not require a 3′ overhang [74]. This might be related to the fact that specific structures in the R2 RNA allow the R2 RT to position and guide the exact start of reverse transcription at the cleavage site [36]. In this configuration, primer-template annealing is no longer a requirement to position the primer at the end of the template. Biochemical studies with non-LTR retrotransposon RT from other clades will be necessary to determine, which of these two situations is the rule and the exception.

The current model of L1 retrotransposition, which has been largely inspired by studies on the R2 element, starts with a nick in the target DNA followed by the extension of this nick. Our data indicate that extension by the L1 RNP is efficient on single-stranded DNA substrates, but inefficient when the 3′ OH is embedded in duplex DNA, either at a blunt end or at a 3′ recessed end (Figure 6B and Figure S4A). In contrast, it efficiently initiates reverse transcription on double-stranded DNA molecules ending with a 3′ single-stranded overhang (Figure 7B and Figure S4B). Thus, our results suggest an additional step in the retrotransposition process, which generates a single-stranded 3′ end from a blunt end or from a nick to allow L1 reverse transcription. We envisage two ways in which this 3′ overhang could be established. In the first model, the L1 endonuclease directly generates a double-strand break with staggered cuts instead of acting sequentially on one strand and then on the other strand only after minus strand cDNA synthesis. Consistently, recombinant L1 endonuclease can linearize plasmid DNA *in vitro* [27] and ectopic L1 expression results in the activation of a DNA damage response in cultured cells [75], [76]. In the second model, an unidentified machinery could promote unwinding of the nicked DNA or permit strand-exchange between the duplex DNA and the RNA moiety of the L1 RNP. The ORF1p protein has been proposed to play such a role through its nucleic acid chaperone activity [20], [24]. Indeed, nucleic acid chaperone activities promote reverse transcription in retroviruses and LTR-retrotransposons through several mechanisms, including primer annealing to the template RNA [77]–[80]. All the experiments described here use native L1 RNP preparations, which contain ORF1p (Figure 1 and Figure 8). However, in our experimental conditions, we were unable to detect extension of blunt or 3′ recessed double-stranded substrates. Thus, if such a DNA remodeling machinery is involved, it has to be of cellular origin. Nevertheless, it should be noted that, in primer extension assays, as performed in LEAP or DLEA experiments, the initiation of reverse transcription is uncoupled from the cleavage of the target DNA, in contrast to the TPRT process. Thus, we cannot completely exclude that the L1 RNP would utilize a different priming mechanism in the context of a L1 TPRT reaction.

The requirement of a 3′ overhang could also be relevant to alternative L1 integration pathways. Indeed, L1s can initiate reverse transcription at preformed DNA lesions or at telomeric ends and thus insert into the genome independently of their EN activity [51], [68]–[70]. EN-independent retrotransposition was only observed in cell lines deficient in the nonhomologous end-joining (NHEJ) pathway [68]. Interestingly, binding of NHEJ components to DNA ends interferes with end resection [81]. As a result of this competition, end resection (the first step of homologous recombination) is increased in NHEJ-deficient cell lines. Thus, we speculate that EN-independent retrotransposition might require the 5′ to 3′ end resection step, which initiates HR, to generate a 3′ overhang suitable for L1 reverse transcription initiation. The link between end resection factors (such as the MRN complex, CtIP, Exo1, BLM, Dna2, etc.) and the ability of L1 to engage in EN-independent insertions will be an important direction for future studies. Similarly, the L1 RNP is also able to prime cDNA synthesis at dysfunctional telomeres in NHEJ-deficient hamster cells [51], [69]. Telomeres end with a 3′ overhang [82], [83], the formation of which is highly regulated and involves a specialized set of factors [84]. Telomeres can also be extended by a specialized cellular RNP with reverse transcriptase activity, called telomerase [85], [86]. Like L1, telomerase requires a 3′ single-stranded overhang to extend double-stranded DNA [87]. Thus our observations reinforce the notion that these two endogenous reverse transcriptases, which are evolutionary related [88]–[90], share common mechanistic properties [51].

In conclusion, our data demonstrate that partial sequence complementarity between the target site and the L1 RNA facilitates L1 reverse transcription priming and highlight the flexibility of the L1 RT. Interestingly, EN cleavage and RT priming appear to target the same TTTT sequence, suggesting that these two L1 biochemical activities have co-evolved. We speculate that their exceptional flexibility has participated in the evolutionary success of the L1 family and in its wide spread distribution within mammalian genomes.

## Materials and Methods

### Plasmids and oligonucleotides

Plasmids JM101/L1.3 and JM105/L1.3 respectively contain WT and RT-mutated (D702A) versions of the human L1.3 element in a pCEP4 backbone (a kind gift of N. Gilbert) [9]. Plasmid pWA121 contains a codon-optimized version of the mouse L1spa element in a pCEP4-Puro backbone (a kind gift of J. D. Boeke) [91]. A fragment containing mORF2p was amplified by PCR from pWA121 using oligonucleotides LOU266 and LOU267. The purified attB PCR product was cloned into pDONR207 using BP Clonase II under the manufacturer's conditions (Gateway system, Life Technologies) to obtain plasmid pVan239. A point mutation in the RT domain (D709A) was introduced in this construct using the QuikChange II XL Site-Directed Mutagenesis Kit (Agilent Technologies) and the DNA primer pair LOU419-LOU420 to generate pVan330 (mORFeus RT*). The RT* mutation introduces a new SacII restriction site in ORF2, allowing quick screening of the mutation. The latter was confirmed by sequencing. A SdaI-NruI DNA fragment containing part of ORF2p from this entry clone was inserted back into the original pWA121 plasmid digested by the same enzymes. A full list of the oligonucleotides used in this study is provided as Table S1.

### Antibodies

Peptides corresponding to the C-termini of mouse (N-CNQYKNGNNALEKTRR-C) or human (N-CERNNRYQPLQNHAKM-C) ORF1p were synthesized and coupled to the KLH protein as a carrier. The first cysteine (underlined) is not present in the ORF1p sequence but was added for the coupling reaction with the carrier protein. KLH-coupled peptides were used to immunize rabbits (Eurogentec). For immunoblotting the mORF1p antiserum (SE-0560), the hORF1p antiserum (SE-6798), and the S6 protein antibody (Cell signaling, #2217) were used at a dilution of 1∶2000.

### Oligonucleotide purification

One hundred micrograms of each lyophilized oligonucleotide was dissolved in 10 µl of 98% deionized formamide, 1 mM EDTA, 0.01% (w/v) xylene cyanol and 0.01% (w/v) bromophenol blue and resolved in 10% polyacrylamide-urea denaturing gels. Full length oligonucleotides were visualized by UV shadowing, excised from the gel and eluted overnight at 37°C in 0.3 M sodium acetate, 0.1% SDS and 10 mM MgCl_2_. Eluted oligonucleotides were precipitated with ice-cold ethanol (3v). After centrifugation for 30 min at 4°C at 16'000 g, the pellets were washed with 70% ethanol, air-dried and dissolved in 10 mM Tris-HCl pH 8.0, 1 mM EDTA.

### Production of L1 RNPs in human cells

L1 RNPs were produced in HEK293T cells grown in Dulbecco's Modified Eagle Medium (DMEM, Life Technologies) containing 2 mM L-Glutamine, 4500 mg/L D-Glucose, 1 mM Sodium Pyruvate, 10% (v/v) fetal bovine serum (Life Technologies) and 100 units/mL penicillin/streptomycin (Life Technologies). Cells were plated at 3×10^6^ cells per 10 cm Petri dish. Twenty-four hours after plating, the cells were transfected with 24 µg of plasmid DNA (see plasmids above) per dish using the calcium phosphate method. Growth medium was changed 5 hours later. One day post-transfection, cells were split into two plates in growth medium supplemented with 1.5 µg/mL puromycin (mORFeus, Life Technologies) or 100 µg/mL hygromycin (L1.3, Life Technologies). Cells were collected 4 days post-transfection by trypsinization, pooled and washed in PBS. Cell pellets were lysed in 500 µL of CHAPS lysis buffer (10 mM Tris-HCl [pH 7.5], 1 mM MgCl_2_, 1 mM EGTA, 0.5% (w/v) CHAPS, 10% (v/v) Glycerol, supplemented before use with Complete EDTA-free protease inhibitors cocktail (Roche) and 1 mM DTT). After incubation at 4°C for 15 min, cell debris was removed by spinning down extracts at 4°C for 10 min at 16'000 g. Supernatants were transferred to clean tubes and 500 µL of lysis buffer were added to each of them.

### Partial purification of L1 RNP by sucrose cushion and ultracentrifugation

L1 RNPs were prepared as previously described [10]. In brief, a sucrose cushion was prepared with 8.5% and 17% (w/v) sucrose in 20 mM Tris-HCl [pH 7.5], 80 mM NaCl, 5 mM MgCl_2_, 1 mM DTT and Complete EDTA-free protease inhibitors cocktail (Roche). For each sucrose cushion, 1 mL of cell lysates, prepared as described above, was used. Samples were centrifuged for 2 h at 178'000 g at 4°C and the pelleted material was resuspended in 100 µL H_2_O. Total protein concentration was determined by Bradford assay (Biorad). The samples were diluted in 50% (v/v) glycerol, quick frozen in liquid nitrogen and stored at −80°C until use.

### Immunoprecipitation of L1 RNP

Protein A-Sepharose beads (Sigma) were blocked overnight at 4°C in PBS containing 0.5 mg/mL of bovine serum albumin (BSA) and washed twice in 1 mL of IP buffer (10 mM Tris-HCl [pH 7.5], 150 mM NaCl). Eight microliters of preimmune or anti-mORF1p serum were bound to 70 µl of blocked beads for 3 h at 4°C. For each immunoprecipitation, 200 µL of L1 RNPs (2 µg/µL) were diluted 1∶1 (v/v) in IP buffer. The RNPs were precleared with blocked beads for 1 h at 4°C and incubated for 3 h at 4°C with antibody-bound beads on a rotating wheel. After 4 washes in IP buffer, the bead slurry was split equally into 7 tubes (6 for RT reactions and 1 for immunoblotting). Beads were pelleted for 5 min at 4°C at 750 g, supernatants were removed and the RT reaction mixture was directly added to the beads (see below).

### Direct L1 extension assay (DLEA)

Reverse transcriptase assays were carried out for 4 min at 37°C in 25 µL reactions containing 2 µg of RNPs, 400 nM of primer, 50 mM Tris-HCl [pH 7.5], 50 mM KCl, 5 mM MgCl_2_, 10 mM DTT, 0.05% (v/v) Tween-20 and 10 µCi of α-^32^P-dTTP (3000 Ci/mmol, PerkinElmer). In reactions using the Avian Myeloblastosis Virus RT (AMV RT, Promega), the RNPs were replaced by 0.04 U of AMV RT and 250 ng of poly(rA) template (Roche). Reactions were stopped by the addition of 8.3 mM EDTA and 0.83% SDS final. Trace amounts of a ^32^P-labelled 14- or 30-mer DNA oligonucleotide were added as recovery control (noted RC (14) or RC (30) in the figures). Products were purified by phenol-chloroform extraction and ethanol precipitation with 10 µg of glycogen as a carrier and 0.1 mM sodium acetate [pH 5.2]. DNA pellets were resuspended in 98% deionized formamide containing 10 mM EDTA, 0.02% (w/v) xylene cyanol and 0.02% (w/v) bromophenol blue, heated to 95°C for 5 min, and analyzed on 13% polyacrylamide-urea sequencing gels. After drying, gels were exposed to a PhosphorImager screen.

For primers used in Figure 4, we first resolved the products on sequencing gels to verify that the profiles of the products were similar to those obtained with other linear oligonucleotides and that nonspecific products were not generated. In a second time, to facilitate quantification of a large number of reactions performed in parallel, we spotted 5 µL of each reaction onto DE-81 paper immediately after the 4 min incubation, in triplicate. DE-81 paper is an ion exchange paper, which retains the incorporated nucleotides, but not the free dNTPs. Papers were next washed 5 times with 200 mL of 2x saline-sodium citrate (SSC) solution and exposed to a PhosphorImager screen. We tested the complete set of primers three times.

For gel or spot quantification, the reaction without primer obtained with a given RNP preparation was used as background and was subtracted from the reaction with primers. Only the signal above the primer size was quantified for the hairpin oligonucleotides.

### RNase treatment and reverse transcriptase inhibitors

To determine whether ^32^P incorporation was RNase sensitive (Figure S1A), we incubated reaction mixes in the presence of 30 µg of RNase A and 150 U of RNase I (New England BioLabs), or of 40 U of RNasin (Promega) as a negative control, for 1 h at 37°C before adding ^32^P-dTTP and primer. RT inhibitors (AZT and d4T, also known as Stavudin) as triphosphate derivatives were obtained from Biocentric. They were added to reactions at a final concentration of 10 µM (Figure S1B).

### L1 element amplification protocol (LEAP)

LEAP was performed as previously described [10] with only minor modifications. Briefly, L1 reverse transcription was carried out for 1 h at 37°C in 50 µL reactions containing 0.75 µg L1 RNP (50% (v/v) glycerol), 50 mM Tris-HCl [pH 7.5], 50 mM KCl, 10 mM DTT, 5 mM MgCl_2_, 0.05% (v/v) Tween-20, 20 U RNasin (Promega), 200 µM dNTP, and 0.4 µM LEAP primer. Eventually, unextended primers were eliminated through an S-400HR size-exclusion spin column (GE Healthcare). Reverse transcription products (1 µL of the LEAP reaction) were PCR-amplified in 50 µL reactions containing 1 U of Platinum Taq DNA Polymerase (Life technologies), 0.2 µM of primers LOU851 and LOU312, 200 µM dNTP, 3 mM MgCl_2_ in the Platinum Taq buffer. A first step at 94°C for 2 min was followed by 35 cycles of [30 s at 94°C, 30 s at 60°C and 30 s at 72°C]. The final extension was at 72°C for 5 min. PCR products were analyzed by 2% agarose gel electrophoresis in 1x TBE. Gels were stained by SYBR Safe (Life technologies) or ethidium bromide. LEAP products were gel-purified with a gel extraction kit (Macherey Nagel) and cloned into the pGEM-T-easy vector (Promega), according to manufacturer's protocol. Clones from isolated colonies were sequenced by GATC. Regions with low quality (Phred<Q20) were trimmed or filtered out using Geneious 5.

### RNA isolation and conventional RT–PCR

Total RNA was extracted from 30 µg of L1 RNP using TRIzol extraction (Molecular Research Center Inc) following the manufacturer's instruction. RNA was resuspended in 20 µL of milliQ water and quantified by Nanodrop. One microgram of RNA was digested by 1 U of RNase-free RQ1 DNase (Promega) in 10 µL reaction in the manufacturer's buffer at 37°C for 30 min. DNase was heat-inactivated for 10 min at 65°C. Then, cDNA synthesis was performed at 50°C for 1 h in 20 µL reactions containing 6 µL of the DNase reaction, 200 U of SuperScript III Reverse Transcriptase (Life technologies), 500 µM dNTP, 50 pmol of RACE primer, 40 U RNAseOUT (Life technologies), 50 mM Tris-HCl [pH 8.0], 75 mM KCl, 3 mM MgCl_2_ and 5 mM DTT. Primer pairs used for PCR were LOU851/LOU312 (mOrfeus or L1.3) or LOU852/LOU312 (GAPDH). PCR products were resolved by 2% agarose gel electrophoresis in 1x TBE.

### T-density and position-weighted T-density

The *T-density* is calculated by dividing the number of Ts in the oligonucleotide by the length of the oligonucleotide. The *position-weighted T-density* gives more weight to Ts which are close the 3′ extremity of the primer. The weight is inversely proportional to the distance from the 3′ end.

For example:

Primer LOU519 has a *position-weighted T-count* equal to:
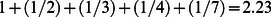



Primer LOU541 has a *position-weighted T-count* equal to:
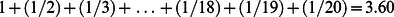



The *position-weighted T-density* of a given primer is calculated by dividing the *position-weighted T-count* of this primer to the maximum *position-weighted T-count*. Thus the *position-weighted T-density* of LOU519 is equal to 2.23/3.60 = 0.62 and the position-weighted T-density of LOU541 is equal to 3.60/3.60 = 1

### Snap and velcro definitions

The snap is considered open if the 4 terminal nucleotides contain a non-T nucleotides and closed if the last four nucleotides are 4 Ts. We calculated a *position-weighted T-count* for the upstream 6 nucleotides (velcro region) and we divided it by the maximum value (1/5)+(1/6)+…+(1/10) = 0.84563492 to obtain the velcro *position-weighted T-density*. We consider a velcro as fastened if its *position-weighted T-density* is ≥0.5 (half of the maximum) and opened otherwise.

### Analysis of snap/velcro category enrichment in genomic datasets

All putative integration sites with a perfect or degenerate EN recognition sequence (from 3′ to 5′, R/TTTT, R/VTTT, R/TVTT, R/TTVT, R/TTTV) were recovered from both strands of the reference human genome (hg19) or from its repeatmasked version (hg19 RM). For each putative EN site, snap and velcro status were defined as described above. The C++ program used to achieve this task is available in Protocol S1. Polymorphic L1 insertions were extracted from dbRIP [59] or from cancer genome whole-genome sequences [60], [61]. Only insertion sites with an identifiable EN recognition site as defined above were kept for the analysis. This filtering step was necessary to eliminate internal initiation events most likely related to EN-independent insertions or other forms of structural variation and insertion sites which position was not precise at nucleotide resolution. Raw data are provided in Table S2. For each dataset, we calculated the frequency of each category and we normalized first to hg19 count and second to the “open snap/tightly fastened velcro” category to evaluate the effect of a closed snap and/or velcro. We compared observed (polymorphic L1 insertions) and expected (hg19) frequencies by Chi-squared test. We used the Graphpad Prism 6.00 software for Mac for all statistical analyses.

## Supporting Information

Figure S1 Additional characterization of the L1 RNP RT activity by DLEA. (A) RNA-dependent DNA polymerase activity of L1 RNPs. Murine L1 RNPs were incubated for 1 h at 37°C in the presence (lane 3) or in the absence (lane 4) of RNases before the start of the reaction. (B) RT inhibitors prevent primer extension by L1 RNPs. Reactions were performed with mL1 RNPs in the presence of thymidine analogs (10 µM of azidothymidine triphosphate AZTTP, denoted by A, lane 3; 10 µM of 2,3-didehydro-3-deoxythymidine triphosphate d4TTP, denoted by D, lane 4), or in the presence of water as a negative control (lane 2). (C) Time-course of (dT)_18_ primer extension by hL1 RNP. (D) Formation of long cDNA species upon addition of all four dNTPs. Reactions were performed with hL1 RNPs in presence of α-^32^P-dTTP and a (dT)_18_ primer, with (lanes 3 & 6) or without (lanes 1–2 & 4–5) cold dATP, dCTP and dGTP (dVTP, IUPAC nomenclature).
(TIF)

Figure S2 The murine L1 RNP preferentially extends primers ending with at least 4 Ts. DLEA showing the extension of single-stranded primers by mL1 RNPs in the presence of α-^32^P-dTTP. RC denotes a 14 nt recovery control added after the reaction but before DNA purification. The black dots on the left side of each lane indicate the expected start of reverse transcription. Their position varies since primer length varies. Quantification of primer extension (% Activity) was relative to levels of extension obtained with oligo(dT)_18_. Primers are identical to Figure 2.
(TIF)

Figure S3 LEAP with hL1 RNPs and mismatched primers. (A) Primers with terminal mismatches. LEAP was performed with RNPs prepared from hL1-transfected cells (top panel), from vector-transfected cells (middle panel), or without RNPs (bottom panel). Primers are identical to those used in Figure 2, except that they have a 5′ extension to anchor the PCR (see Table S1 for sequence). (B) Primers mimicking L1 integration sites. LEAP was performed with RNPs prepared from hL1-transfected cells (top panel), from vector-transfected cells (middle panel), or without RNPs (bottom panel). Primers are identical to those used in Figure 4, except that they have a 5′ extension to anchor the PCR (see Table S1 for sequence). (C) LEAP products from (A) and (B) were gel purified, cloned and sequenced. For each oligonucleotide, the top sequence and number of clones correspond to the extension of unprocessed primer, whereas other sequences correspond to the extension of processed primers.
(TIF)

Figure S4 Human L1 RNPs preferentially extends double-stranded DNA with a 3′ overhang. (A) Absence of extension by hL1 RNPs of double-stranded primers with blunt or 3′-recessed end in the presence of α-^32^P-dTTP. Note that the products observed with hairpin primers (lanes 3–7) result from contaminating cellular activities (see main text and Figure 8). (B) Extension by hL1 RNPs of double-stranded primers ending with a 3′ overhang in the presence of α-^32^P-dTTP. Note that the doublet below 40 nt observed in lanes 3–11 and 14–22 results from contaminating cellular activities (see text and Figure 8 for further characterization). RC denotes a 30 nt recovery control added after the reaction but before DNA purification. The black dots on the left side of each lane indicate the expected start of reverse transcription. Their position varies since primer length varies. Results obtained with mL1 RNPs were identical and are shown in Figure 6, Figure 7, Figure 8.
(TIF)

Protocol S1 Source code of the software used to find putative endonuclease sites in the human genome and to calculate their associated snap/velcro scores.
(GZ) Click here for additional data file.

Table S1 List of oligonucleotides used in this study.
(XLSX)

Table S2 Data used to calculate genomic enrichment of L1 insertions depending on the snap-velcro status of the target. The table sheets are the following: (hg19) For each potential L1 EN target site present in hg19, the snap status was defined and the position-weighted A density was calculated. Sites with position-weighted A density equal to or above 0.5 were considered as having a closed velcro strap. (hg19 RM) Same as above but with a repeatmasked hg19 reference genome. (dbRIP sequences) L1HS dbRIP entries used in Figure 5C and 5C and their snap/velcro status. (dbRIP counts) Number of dbRIP entries in each category. (dbRIP weblogo) Weblogo of the junction sequence (−2/+10) for dbRIP entries. (Lee2012 sequences) L1HS somatic insertions in cancer used in Figure 5C and 5C and their snap/velcro status. (Lee2012 counts) Number of L1HS somatic insertions in each category. (Lee2012 weblogo) Weblogo of the junction sequence (−2/+10) for Lee2012 entries. (Solyom2012 sequences) L1HS somatic insertions in colon cancer used in Figure 5C and 5C and their snap/velcro status. (Solyom2012 counts) Number of L1HS somatic insertions in each category. (Solyom2012 weblogo) Weblogo of the junction sequence (−2/+10) for Solyom2012 entries.
(XLSX)
