# Supplementary figures and images for: The Specificity and Flexibility of L1 Reverse Transcription Priming at Imperfect T-Tracts
Source: PLoS Genet. 2013 May 9;9(5):e1003499. doi: 10.1371/journal.pgen.1003499 (PMC3649969; doi:10.1371/journal.pgen.1003499)

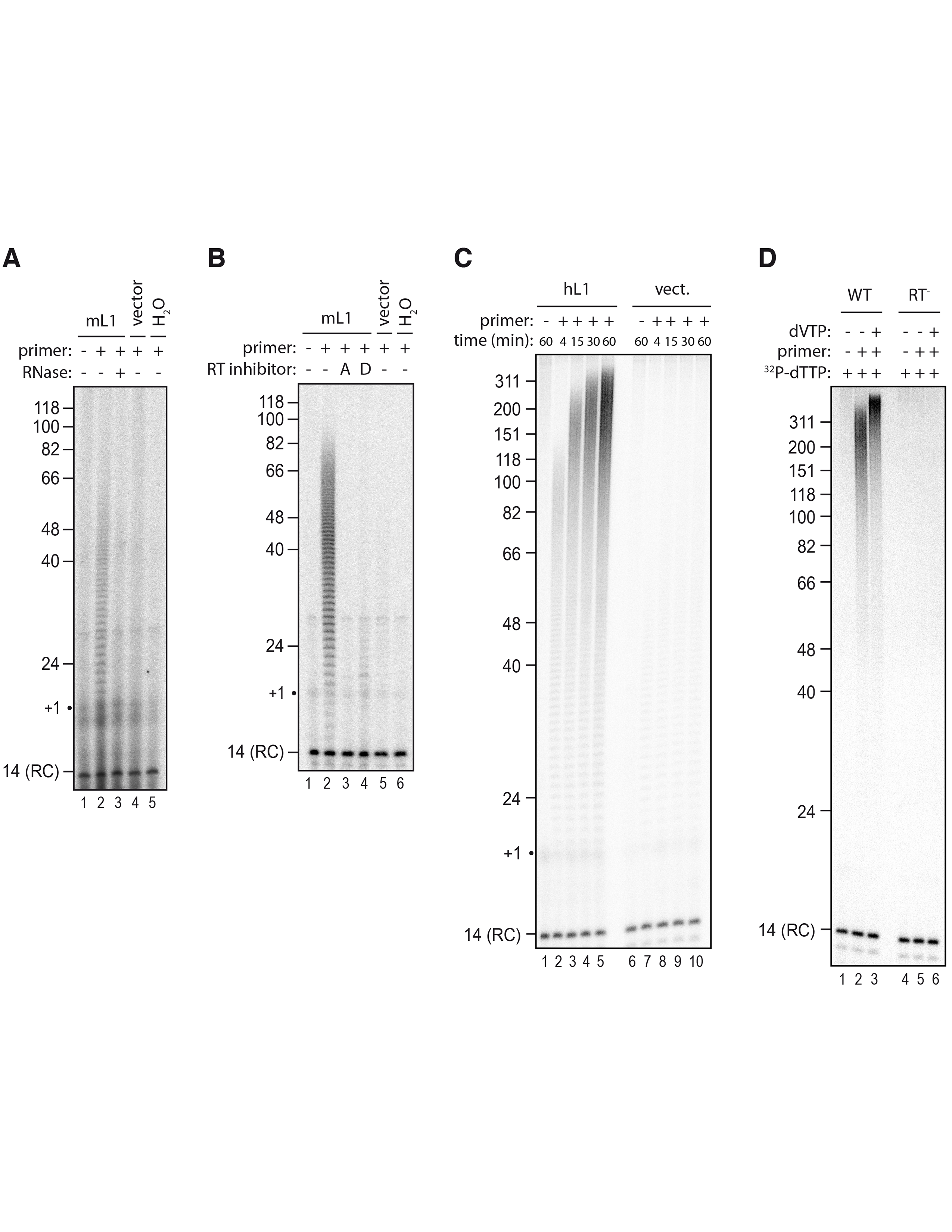

Supplement: Figure S1 — Additional characterization of the L1 RNP RT activity by DLEA. (A) RNA-dependent DNA polymerase activity of L1 RNPs. Murine L1 RNPs were incubated for 1 h at 37°C in the presence (lane 3) or in the absence (lane 4) of RNases before the start of the reaction. (B) RT inhibitors prevent primer extension by L1 RNPs. Reactions were performed with mL1 RNPs in the presence of thymidine analogs (10 µM of azidothymidine triphosphate AZTTP, denoted by A, lane 3; 10 µM of 2,3-didehydro-3-deoxythymidine triphosphate d4TTP, denoted by D, lane 4), or in the presence of water as a negative control (lane 2). (C) Time-course of (dT)18 primer extension by hL1 RNP. (D) Formation of long cDNA species upon addition of all four dNTPs. Reactions were performed with hL1 RNPs in presence of α-32P-dTTP and a (dT)18 primer, with (lanes 3 & 6) or without (lanes 1–2 & 4–5) cold dATP, dCTP and dGTP (dVTP, IUPAC nomenclature). (TIF) [file pgen.1003499.s001.tif]

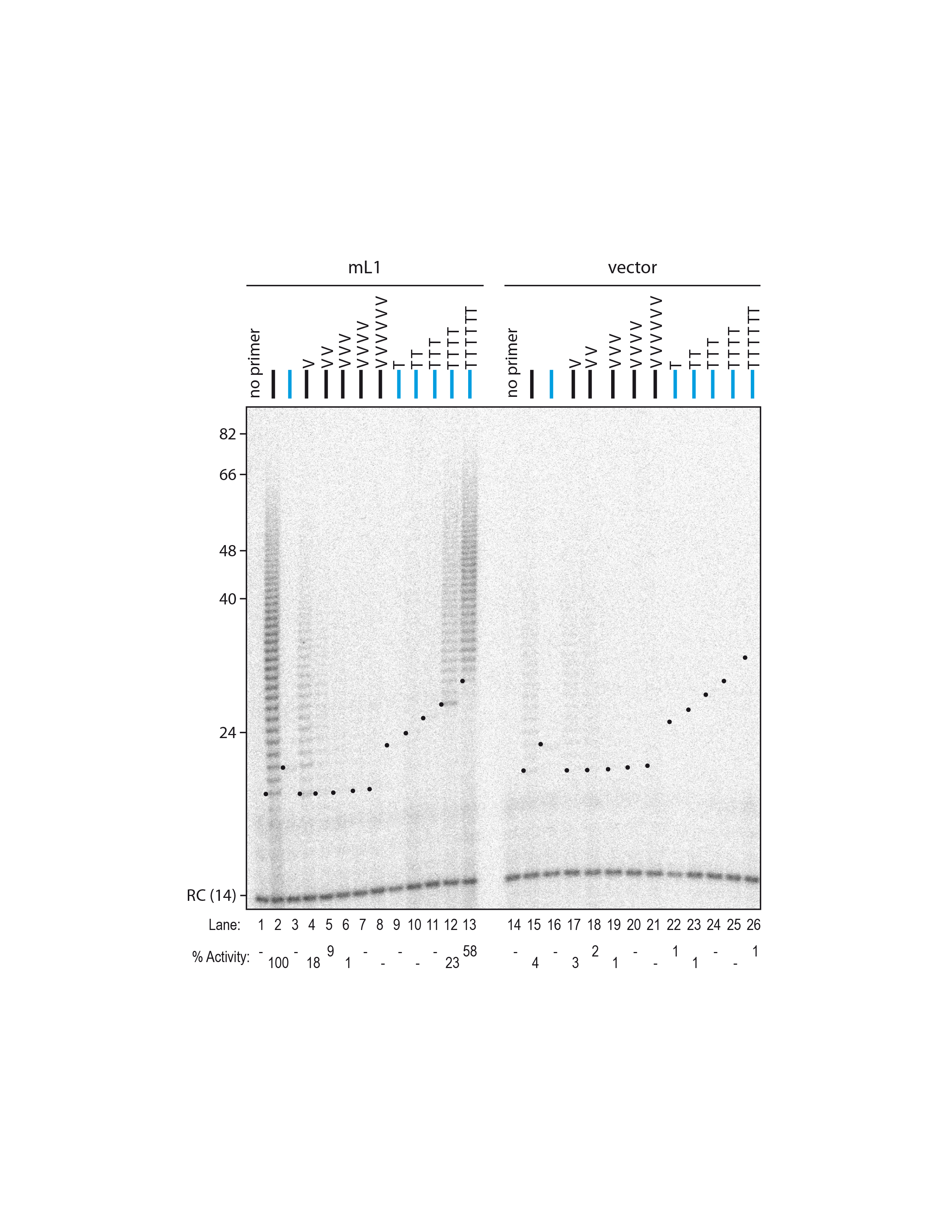

Supplement: Figure S2 — The murine L1 RNP preferentially extends primers ending with at least 4 Ts. DLEA showing the extension of single-stranded primers by mL1 RNPs in the presence of α-32P-dTTP. RC denotes a 14 nt recovery control added after the reaction but before DNA purification. The black dots on the left side of each lane indicate the expected start of reverse transcription. Their position varies since primer length varies. Quantification of primer extension (% Activity) was relative to levels of extension obtained with oligo(dT)18. Primers are identical to Figure 2. (TIF) [file pgen.1003499.s002.tif]

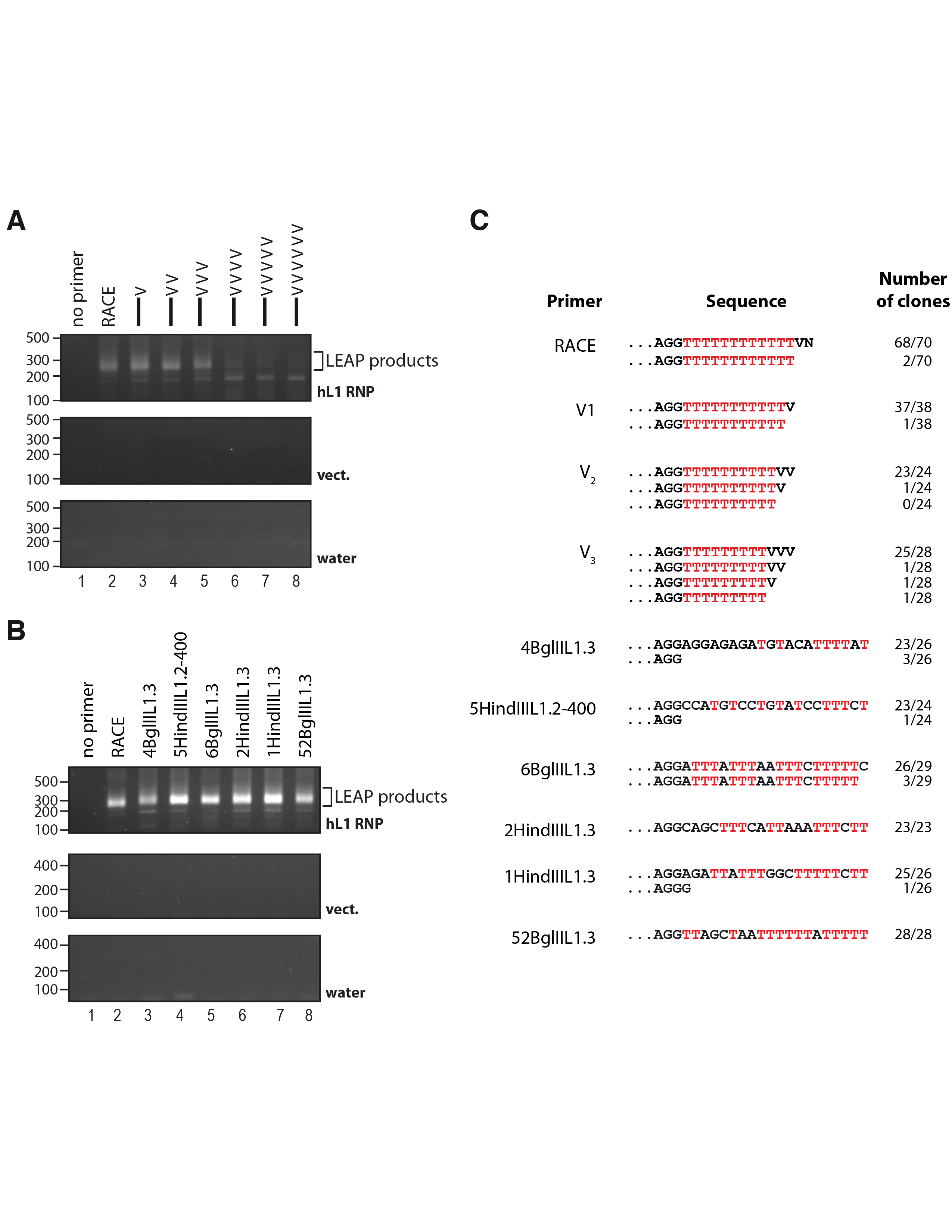

Supplement: Figure S3 — LEAP with hL1 RNPs and mismatched primers. (A) Primers with terminal mismatches. LEAP was performed with RNPs prepared from hL1-transfected cells (top panel), from vector-transfected cells (middle panel), or without RNPs (bottom panel). Primers are identical to those used in Figure 2, except that they have a 5′ extension to anchor the PCR (see Table S1 for sequence). (B) Primers mimicking L1 integration sites. LEAP was performed with RNPs prepared from hL1-transfected cells (top panel), from vector-transfected cells (middle panel), or without RNPs (bottom panel). Primers are identical to those used in Figure 4, except that they have a 5′ extension to anchor the PCR (see Table S1 for sequence). (C) LEAP products from (A) and (B) were gel purified, cloned and sequenced. For each oligonucleotide, the top sequence and number of clones correspond to the extension of unprocessed primer, whereas other sequences correspond to the extension of processed primers. (TIF) [file pgen.1003499.s003.tif]

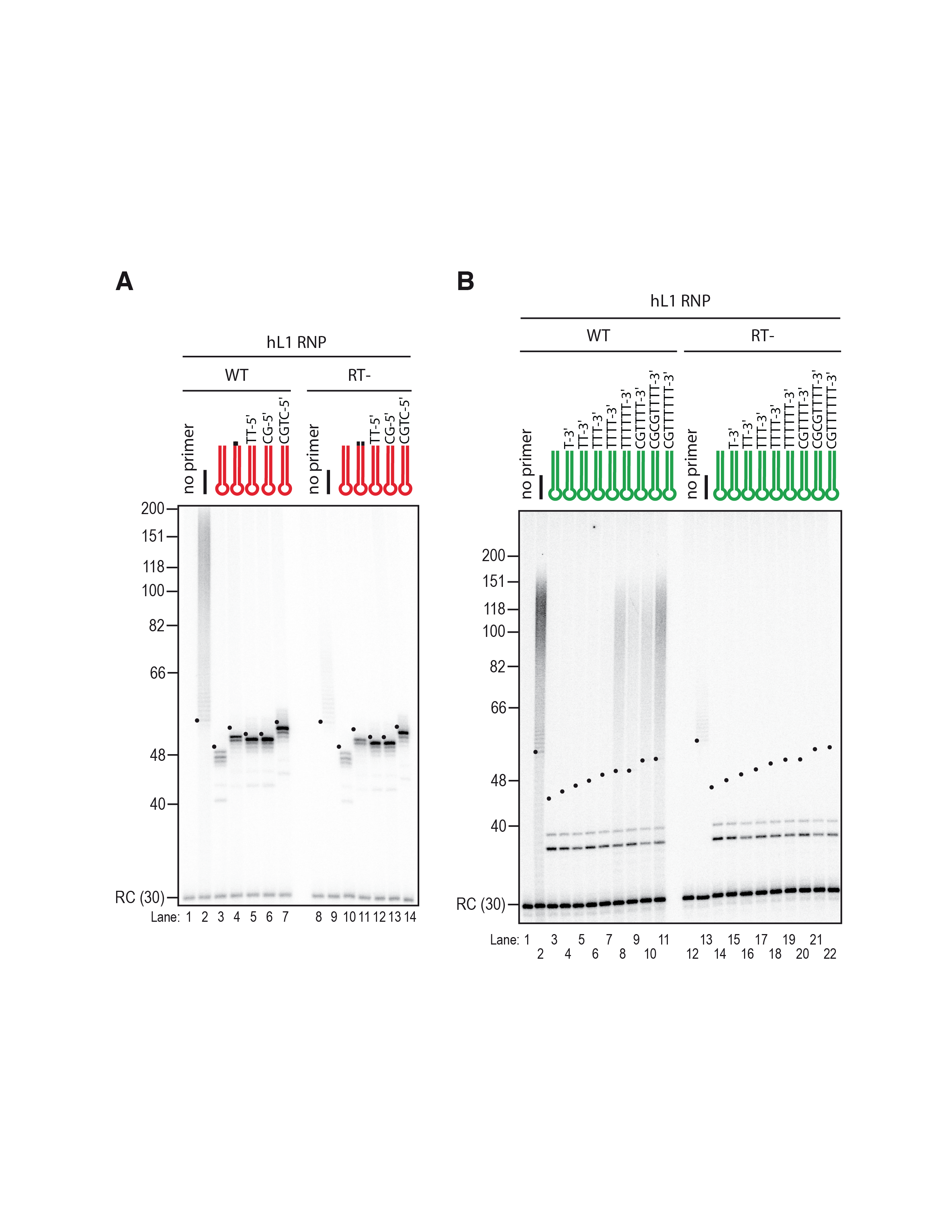

Supplement: Figure S4 — Human L1 RNPs preferentially extends double-stranded DNA with a 3′ overhang. (A) Absence of extension by hL1 RNPs of double-stranded primers with blunt or 3′-recessed end in the presence of α-32P-dTTP. Note that the products observed with hairpin primers (lanes 3–7) result from contaminating cellular activities (see main text and Figure 8). (B) Extension by hL1 RNPs of double-stranded primers ending with a 3′ overhang in the presence of α-32P-dTTP. Note that the doublet below 40 nt observed in lanes 3–11 and 14–22 results from contaminating cellular activities (see text and Figure 8 for further characterization). RC denotes a 30 nt recovery control added after the reaction but before DNA purification. The black dots on the left side of each lane indicate the expected start of reverse transcription. Their position varies since primer length varies. Results obtained with mL1 RNPs were identical and are shown in Figure 6, Figure 7, Figure 8. (TIF) [file pgen.1003499.s004.tif]
